# Supplementary material for: Lithiated porous silicon nanowires stimulate periodontal regeneration
Source: Nat Commun. 2024 Jan 12;15:487. doi: 10.1038/s41467-023-44581-5 (PMC10786831; doi:10.1038/s41467-023-44581-5)
Supplement: Supplementary file 1 — Supplementary Information [file 41467_2023_44581_MOESM1_ESM.pdf]

Supplementary Information

Lithiated porous silicon nanowires stimulate periodontal regeneration

Table of Contents

1 Materials and methods *2*
1.1 Metal assisted chemical etching (MACE) 2
1.2 Lithiation 2
1.3 Nanowires sizing with SEM 3
1.4 Nanowire composition and release kinetics 3
1.5 ICP-MS measurements 3
1.6 Nanowire sizing and composition with CF3-MALS-ICPMS 3
1.7 Surface area and porosity with gas sorption 4
1.8 HRTEM and EELS analysis 4
1.9 XRPD analysis 4
1.10 Raman Microspectroscopy 4
1.11 MAS-NMR Analysis 5
1.12 XPS analysis 5
1.13 Synchrotron XANES measurements 5
1.14 Preparation of conditioned medium 5
1.15 ATP activity and Wnt pathway activation of human PDL cells 6
1.16 Osteogenic differentiation assay 7
1.17 Preparation of fluorescently-labelled nanowires 8
1.18 Nanowire distribution following PDL injection 8
1.19 Evaluation of Wnt/β-catenin pathway activation following PDL injection 8
1.20 Evaluation of LipSi activity in periodontal defect *in vivo* 9
1.20.1 Animals and surgical procedures 9
1.20.2 µCT analysis 9
1.20.3 Histological analyses and multiplex immunofluorescent staining 9
1.21 LA-ICP-MS measurements 10
2 Supplementary Figures *11*

1 Materials and methods

1.1 Metal assisted chemical etching (MACE)

Porous silicon nanowires were etched via metal assisted chemical etching (MACE) as described in our previous publications[1]. A schematic representation of the MACE process can be found in Figure 1a. Briefly, prime grade 100 mm silicon wafers, with boron doping, 0.01-0.02 Ωcm resistivity, and (100) orientation, were purchased from University Wafers Inc (USA). Wafers were first cleaned from native surface oxide by dipping them into 1:4 mixture of hydrofluoric acid 50 % (Honeywell 40213H) and deionised (DI) water (10 % HF). Cleaned wafers were rinsed with DI water and isopropanol and dried in N_2 flow. After cleaning, wafers were immersed into solution of 20 mM silver nitrate ($AgNO_3$, Sigma-Aldrich 99.9999%) in 10 % HF for 2 minutes under constant gentle stirring. Wafers were rinsed and dried as before. Etching of porous silicon nanowires was done by immersing silver deposited wafers for 20 minutes in an etching solution containing 0.6 wt% (or 1.2 wt%) of H_2O_2 (Honeywell, 95299) in 10% HF. Etched wafers were rinsed and dried as before. As a last step, deposited silver particles were removed by 10 min treatment with standard gold etchant (Aldrich, 651818). Etched wafers were rinsed and dried as before. Prior the use, nanowires were collected by scraping with razor blade.

1.2 Lithiation

Schematic representation of the lithiation process can be found in Figure 1b. Porous silicon nanowire powder was mixed with lithium containing agents: LiCl (Fluorochem, 091451), LiOH*H_2O (Fluorochem, 009861) or Li_2CO_3 (Acros Organics, 197785000). Two different mixing processes were used, grinding for LiOH and Li_2CO_3 or liquid evaporation for LiCl. In the grinding method, a pre-weighted pSi nanowire powder (brown) and solid lithium source (white) were combined in a mortar (Supplementary Figure 2). The particle size of the lithium source and agglomerate size of pSi nanowires was reduced by gently grinding with a pestle. Mixing/grinding was done until the powder appeared homogenous in structure and colour. The powder was then poured on top of a silicon wafer, covered with a steel vial, and moved on a hotplate preheated to 450 °C. In the solvent evaporation method, LiCl was dissolved into methanol at 40 mg/ml concentration. pSi nanowire powder and LiCl solution were combined in mortar, mixed briefly with the pestle, and moved to a hotplate preheated to 100 °C. After the methanol had evaporated, the temperature was increased to 150 °C for 10 minutes to enable evaporation of any remaining solvent or moisture. The solid mixture of pSi and LiCl was moved onto a Si wafer preheated to 150 °C and made into a fine powder. The Si wafer was moved onto a hotplate preheated to 450 °C and covered immediately with a bell jar. If treatment was performed in ambient atmosphere, the top cap was left open, if the treatment was performed in N_2 atmosphere, N_2 flow was directed into bell jar through the top valve.

After lithiation, the excess lithium precursor was washed by dispersing the nanowires into dilute 1mM HCl (in case of the LiOH precursor 1M HCl was used), mixed with a sonicator bath, centrifuged for 8 minutes at 18 000 x g, and the supernatant was then discarded. The washing cycle was repeated 4 times with different media (HCl, H_2O, isopropanol, isopropanol). After the final wash, nanowires were dispersed into isopropanol with a sonicator bath and stored at RT.

The concentration of the LipSi suspension was determined by weighting the solid content. 0.5 ml of LipSi suspension was pipetted into a pre-weighted 1.5ml microcentrifuge tubes, nanowires were spun down, isopropanol was discarded and the tubes were dried at 60 °C for at least 30 min. The caps were closed and the tubes were allowed to reach the original temperature and moisture level by keeping them at RT for at least 15 minutes prior weighing again. At least two microcentrifuge tubes were use per one sample and the weighing was performed at least 3 times.

1.3 Nanowires sizing with SEM

Nanowires were spotted on a clean flat silicon surface from isopropanol suspension in a 10 µL volume. The nanowires were dried and imaged by in-lens detector in a Zeiss XB1540 SEM (Carl Zeiss AG), with voltage 10 keV. Secondary electron micrographs in Figure 1a were obtained with Apreo S field emission SEM (Thermo Scientific Inc.).

1.4 Nanowire composition and release kinetics

The relative content lithium and silicon within LipSiNs was determined with inductively coupled plasma mass spectroscopy (ICPMS) measurements. A known mass of LipSi nanowires were pipetted into a 1.5 ml microcentrifuge tube, centrifuged at 18000 x g for 10 min, the supernatant was discarded, and the pellet was dried at 60 °C for 30 min. The tubes were filled with 1M KOH, known to rapidly dissolve porous silicon[2], to achieve a solid concentration of 100 µg/ml. The nanowires were dispersed by sonication and left at RT for 11 days by shaking every 3rd or 4th day. Following the 11 days of incubation, all samples looked clear. The tubes were centrifuged at 18000 x g for 10 min and 100 µl sample was drawn from the supernatant even though no clear pellet was observed. The samples were diluted into 1.9 ml of 1 % HNO_3 and sent to ICP-MS measurement.

Lithium and silicon release in pH 7.4 phosphate buffer (PB) was studied and the ion concentration was measured with ICP-MS. 200 µg of LipSi nanowires were dried in a 5 ml microcentrifuge tube as described before. 4 ml of pre-warmed 50 mM PB was added and nanowires were dispersed by sonication. Tubes were placed in a thermoshaker (Eppendorf ThermoMixer C) at 37 °C and 650 RPM. At predetermined time points, tubes were centrifuged at 18000 RPM for 10 min and 0.4 ml sample was drawn from supernatant. 0.4 ml of fresh pre-warmed PB was added, the pellet was briefly sonicated, and tubes were placed back in the thermoshaker. All 0.4 ml samples were stored at RT until the experiment was concluded after which they were diluted with 1.6 ml of 1 % HNO3 and sent to ICP-MS measurement. The data was normalised to the last measurement point.

Release studies were also performed in Fusayama/Meyer Artificial Saliva (pH 4.9; Sigma Aldrich) at 37°C and the concentrations were measured at different points with ICP-MS. The initial amount of LipSi nanowires (i.e. 200 µg) and the methodology was followed as described above for release in PB.

1.5 ICP-MS measurements

Inductively coupled plasma mass spectrometry (ICP-MS) analysis of Li (m/z 7) and Si (m/z 28) was performed in either a PerkinElmer NexION 350D or NexION 2000B. The instrument was setup in for KED (kinetic energy discrimination) to reduce problematic interference on the analyte masses, where helium was used as the cell gas. Internal standards were used: Rh was paired with Si and In with Li. Prior to the measurements 2mL of sample was diluted to 10mL. The internal standards were added on-line to the instrument by an Elemental Scientific prepFAST autosampler which also conducted the final stage dilutions (5x) for the samples.

1.6 Nanowire sizing and composition with CF3-MALS-ICPMS

Centrifugal field-flow fractionation (CF3, Postnova CF2000) coupled with UV spectroscopy (UV, Postnova, PN3211), dynamic light scattering (DLS, Malvern Zetasizer Nano), multiangle light scattering (MALS, Postnova PN3621) and inductively-coupled plasma mass spectrometry (ICPMS, Agilent 7900) was used to evaluate the size distribution of the nanowires and the distribution of lithium across the different size fractions of nanowires.

All samples were diluted with ultrapure water (UPW) adjusted to pH 5.2 with nitric acid. The same medium was used as eluent. The final concentration of LipSiN suspensions were between 0.30 and 0.83 mg/ml. Samples were sonicated with probe sonicator for 10 min with 70% power and 50% on/off cycling. After this samples were placed in the autosampler at RT. Injection volumes were 50, 80, 30 and 100 µl for pSi, LipSi-5%, and LipSi-

S4%, respectively and eluent flow rate was 0.5 ml/min. After CF3 (250 µm channel height) fractionation, the stream was monitored with detectors of UV absorption at 254 nm, MALS, DLS and finally ICP-MS.

1.7 Surface area and porosity with gas sorption

LipSi and pSi suspensions were dried in 60 °C and the dry powder was transferred into the measurement tube. Powders were prepared in vacuum at 60 °C for two hours and, after mass measurement, the tubes were attached to the instrument. The porosity of the samples was characterized by N_2 sorption at -196 °C using TriStar 3000 (Micromeritics Inc.). In addition to N_2 sorption, Kr adsorption measurements at -196 °C using 3Flex 3500 (Micromeritics Inc.) were done to complement the surface area determinations. Specific surface areas (SSA) were calculated using the Brunauer-Emmett-Teller (BET) method. Pore volume (V_p) was estimated with Density Functional Theory (DFT) calculations from the N_2 sorption isotherm using silica-based model for cylindrical pores (MicroActive v.5.01, Micromeritics Inc.). Porosity was calculated using the formula

$$P = \frac{V_p}{V_p + \frac{1}{\rho_{Si}}},$$

where $\rho_{Si} = 2.33\ g/cm^3$.

1.8 HRTEM and EELS analysis

LipSi and pSi suspensions were centrifuged at 21 000 x g in 5 ml Eppendorf tubes and dried at 70 °C for 2h. The suspensions were drop-cast onto holey carbon copper grids. The data was collected on an FEI Titan^3 Themis G2 operated at an accelerating voltage of 300 kV, equipped with a field emission gun (X-FEG) operating at an extraction voltage of 4.5 kV and a monochromator. High resolution transmission electron microscopy images and selected area electron diffraction patterns were collected on a Gatan OneView CMOS camera.

STEM-EELS data were collected using a Gatan Quantum 965 ER energy filter for dual EELS (allowing for core-loss edges to be calibrated against simultaneously acquired low-loss spectra). A 0.1 eV/channel dispersion and 5mm entrance aperture were used with approximate energy resolution of 2 eV. The collection and convergence semi-angles were 41.8 and 8.2 mrad respectively. Collected spectra were processed in Gatan Microscopy Suite by aligning the spectra to the zero-loss peak and then applying a power law background subtraction. The relative thickness was calculated by taking the ratio of the zero-loss peak to the plasmon peak from the low-loss spectra.

1.9 XRPD analysis

The crystal structure of LipSi and pSi was evaluated using X-ray powder diffraction (XRPD). Dried LipSi and pSi nanowires were placed on a zero diffraction plate and measured with Empyrean diffractometer (Malvern Panalytical Ltd.) using Cu Kα radiation. The diffractometer was operated in θ/θ Bragg-Brentano configuration with 0.04 rad Soller slits and Bragg-Brentano^{HD} incident beam mirror. The diffraction data was collected with PIXcel^{3D} detector in 1D scanning line mode. The obtained diffractograms were analysed using HighScore Plus v4.9 (Malvern Panalytical Ltd.)

1.10 Raman microspectroscopy

The nanowires were spotted onto magnesium fluoride slides (Global Optics, UK), from isopropanol suspension. Multiple droplets were added after previous had been dried to achieve full coverage. The spectra were acquired with a Renishaw InVia Raman micro-spectrometer using a 40x objective with NA = 0.45, 633 nm wavelength laser, 1mW power, 1200 l/mm grating, 1 s exposure time, 15 accumulations. The bright field microscope allowed focusing and selecting 10 regions per sample containing nanowires. For each spectrum, silicon peak at

~520 cm^{-1} was fitted with the software CasaXPS. The position of the silicon peak was determined by system background subtraction and fitting with a Lorentzian peak.

1.11 MAS-NMR Analysis

LipSi suspension was dried at 60 °C and the measurement cylinder was filled with the dry powder. MAS-NRM ^{7}Li and ^{29}Si MAS-NMR spectra were measured on a 400 MHz NMR spectrometer with Bruker Avance console operating at frequency of 194.35 MHz. Single-pulse excitation was used in combination with a recycle delay of 3 s. The sample rotation rate was 12.5 kHz.

1.12 XPS analysis

The chemical composition of the LipSiNs surface was measured with X-ray photoelectron spectroscopy (XPS). XPS Analysis was performed using a Kratos Axis SUPRA XPS fitted with a monochromated Al Kα X-ray source (1486.7 eV), a spherical sector analyser and 3 multichannel resistive plate, 128 channel delay line detectors. All data were recorded at 150W and a spot size of 700 x 300 µm. Survey scans were recorded at a pass energy of 160 eV, and high-resolution scans recorded at a pass energy of 20 eV. Electronic charge neutralization was achieved using a low energy electron flood gun. Filament current = 0.27 A, charge balance = 3.3 V, filament bias = 3.8 V. Sample etching was performed using a GCIS Ar cluster gun, operating using a 1 mm raster scan, Ar_{500}^{+} clusters and an accelerating voltage of 20 kV. All sample data were recorded at a pressure below 10^{-8} Torr and a room temperature of 21 °C. Additional XPS measurements were conducted using Thermo Scientific Nexsa instrument with monochromated Al Kα X-ray source, using a spot size of 400 µm. The survey scans were obtained with a pass energy of 150 eV and the core level scans at a pass energy of 50 eV. Dynamic charge neutralization was done using a combined low-energy electron and Ar ion beam. The data was analysed using CasaXPS versions 2.3.19PR1.0 (Axis SUPRA) and 2.3.25PR1.0 (Nexsa). Peaks were fit with a Shirley background prior to component analysis. Asymmetric Lorentzian lineshapes were used to fit components. Lithium metasilicate (Li_2SiO_3) was used as reference material and was bought from Santa Cruz Biotechnology (Sc-269334)

1.13 Synchrotron XANES measurements

Lithium K-edge and Silicon L-edge X-ray absorption near edge structure (XANES) measurements were performed at VLS-PGM beamline at the Canadian Light Source under proposal #10662. Powdered experimental samples and standards were mounted onto adhesive carbon tape and mounted into the VLS-PGM end station. Standards were acquired from Sigma Aldrich (St. Louis, MO, USA) (LiCl, Li_2SiO_3, Li_2O, $Li_3C_6H_5O_7$, Li_2CO_3) and Gelest (Morrisville, PA, USA) (Li_xSi_y). All samples were transferred into the measurement chamber in ambient conditions, except lithium silicide which was transferred within Ar gas due to its reactivity. Once loaded, measurements were conducted in vacuum. The fluorescent yield (FLY) was acquired for all samples from 47.5 to 75eV for Lithium K-edge and 95 to 121 eV for Silicon L-edge. FLY data was normalised to the maximum intensity and anomalous points were removed which are common due to monochromator artefacts. Data were fitted in software package ATHENA (Demeter, Bruce Ravel) allowing visual comparison between spectra and removes affects caused by variable concentrations.

1.14 Preparation of conditioned medium

In vitro experiments for viability and Axin2 expression were performed using cell culture medium containing DMEM, 10% FBS, 1% penicillin/streptomycin, 1% L-glutamate which was conditioned with nanowires.

A 5 ml microcentrifuge tube was flushed with isopropanol for sterilisation and a known mass of LipSi nanowires was added. The suspension was centrifuged, and the supernatant (isopropanol) was removed. Nanowires were

dried under a laminar flow biosafety cabinet. Cell medium was added to the tube and nanowires were redispersed by sonication. The tubes were sealed with parafilm and incubated in a thermoshaker at 37°C for four days. Suspension was centrifuged (18000 x g for 7+7 minutes) to remove the remaining nanowires, and the supernatant (conditioned medium) was collected under sterile conditions. The conditioned medium was filtered with 0.22 µm syringe filter, 10 vol% of fetal bovine serum (FBS, Gibco 10270106) was added, and medium was stored in the fridge until used.

1.15 ATP activity and Wnt pathway activation of human PDL cells

Primary human PDL cells (hPDLs) used in these assays were a kind donation from Dr Ana Angelova in King's College London and were used at passage 2-3. These cells were expanded in DMEM high glucose, 10% FBS, 1% L-glutamine and 1% penicillin/streptomycin.

For the viability of hPDL after treatment with different concentration of nanowires an ATP based assay using CellTiter-Glo® 2.0 Assay (Promega) was used. hPDL cells were seeded at 2×10^4 cells/cm^2 in 96 well plate and incubated in cell culture incubator (37°C, 5% CO2) for 24 hr. Next cell culture medium was replaced with medium containing nanowires at different concentrations and incubated for another 24 hr. Cell viability was tested by CellTiter-Glo Luminescent Cell Viability Assay following manufacture's instruction and reading the luminescence with a plate reader (BMG Labtech). Reported viability represents the measured viability signal from cells incubated with nanowires normalised to the signal from the cells incubated with conditioned medium only. Surfactant Triton X-100 was used as cytotoxic positive control. Normal distribution of results was tested with Shapiro-Wilk test and the statistical significance against 100% viability in Med was reported using one-way ANOVA and Dunnett's multiple comparisons in GraphPad Prism 8.3.0. Adjusted P value is reported in graphs according to New England Journal of Medicine guidelines: $P < 0.001$ (***), $P < 0.002$ (**), $P < 0.033$ (*), $P > 0.12$ (ns).

For the effect of nanowires on the activation of Wnt pathway in hPDL, cells were seeded in 24 well plate at the density of 5×10^4 cells/cm^2 and incubated for 24 hr. Cell culture media was then replaced with media containing the selected concentration of nanowires (that were not toxic to the cells) and incubated for another 24 hr. 5 mM LiCL was used as a positive control for Wnt pathway activation. Samples were then lysed, and total mRNA was extracted using TRIzol reagent (Invitrogen, 15596026) according to the manufacturer's instructions. The RNA was reverse transcribed using random primers (M-MLV Reverse Transcriptase kit, Promega) according to the manufacturer's instructions. Gene expression was then assayed by real-time qPCR using Kappa SYBR Green (Kappa Biosystems) on a Rotor-Gene Q cycler (Qiagen) system. Reactions were performed in triplicate and relative changes to housekeeping gene (RPL13A) were calculated by the $2^{-\Delta\Delta CT}$ method where C_T is the threshold cycle. Groups were then analysed with one-way ANOVA followed up with Tukey's multiple comparison tests in GraphPad Prism 8. Normal distribution of results was also tested with Shapiro-Wilk test. Adjusted P value is reported in graphs according to New England Journal of Medicine guidelines: $P < 0.001$ (***), $P < 0.002$ (**), $P < 0.033$ (*), $P > 0.12$ (ns). The following primers were used in this assay: 5' AAGTACCAGGCAGTGACAG 3' (RPL13A-F), 5' CCTGTTTCCGTAGECTCATG 3' (RPL13A-R), 5'CAACACCAGGCGGAACGAA 3' (Axin2-F) and 5'GCCCAATAAGGAGTGTAAGGACT 3' (Axin2-R).

A study using 171A4 cells was conducted to confirm the therapeutic effect of the released ions from nanowires (LipSi and pSi) at different concentrations, 17IA4 cells were seeded in 24 wells plate and the quantity of each well was 5×10^4 cells at 37 °C and 5% CO_2, in α-MEM supplemented with 10% (v/v) FBS and 1% penicillin/streptomycin. The nanowires groups we set are 100 µg/ml, 75 µg/ml, 50 µg/ml, 25 µg/ml, 10 µg/ml, 1 µg/ml and the control group (without nanowires).

Total mRNA was extracted using TRIzol reagent according to the manufacturer's instructions. The RNA was reverse transcribed using iScript™ cDNA synthesis kit according to the manufacturer's instructions. Gene expression was then assayed by real-time qPCR using Fast SYBR® Green Master Mix (Qiagen). Reactions were performed in triplicate and relative changes to housekeeping genes (RPL13A and β-actin) were calculated by the $2^{-\Delta\Delta CT}$ method where C_T is the threshold cycle. The tested target genes were Axin2, RUNX2 and BMP2. All

the data were analyzed by GraphPad Prism 9.0 (GraphPad Software, California, USA), the values were expressed as mean ± standard deviation and statistical analysis was done using one-way ANOVA test. *P* values of less than 0.05 were considered statistically significant.

The following primers were used in this assay:

Gene	Forward Primer sequences(5′-3′)	Reverse Primer sequences(5′-3′)
Axin2(H)	CAACACCAGGCGGAACGAA	GCCCAATAAGGAGTGTAAGGACT
RUNX2(H)	TGGTTACTGTCATGGCGGGTA	TCTCAGATCGTTGAACCTTGCTA
BMP2(H)	ACTACCAGAAACGAGTGGGAA	GCATCTGTTCTCGGAAAACCT
β-actin(H)	CACCAACTGGGACGACAT	ACAGCCTGGATAGCAACG
RPL-13A(H)	AAGTACCAGGCAGTGACAG	CCTGTTTCCGTAGCCTCATG

1.16 Osteogenic differentiation assay.

Human periodontal ligament stem cells were provided by the Oral Stem Cell Bank of Beijing, Tason Biotech Co. Ltd characterised according to B-M Seo et al.[3] hPDLSCs were seeded at passage 5 with 8.0 x 10^3 cells in 0.5 ml growth medium [DMEM (Dulbecco's Modified Eagle Medium), High Glucose, 2117117] supplemented with 10% fetal bovine serum (Biological Industries, 2029091), 1% penicillin/streptomycin (Biological Industries, 2052256) and 2mM glutamine (Corning cellgro, R25015017) per well of a 24-well plate. When cells reach 70% confluence, the medium in each well was replaced with 0.5 ml growth medium, pSiN conditioned medium (§ 1.14), LipSiN conditioned medium (§ 1.14) or osteogenic induction medium [DMEM with 10% fetal bovine serum (Biological Industries, 2029091), β-glycerophosphate (Sigma, G9422) 10 mmol/l, ascorbic acid (Sigma, A4402) 50 g/ml, and dexamethasone (sigma, D1756) 1 × 10 mol/l]. The hPDLSCs were cultured in a humidified atmosphere of 5% CO_2 in air at 37 °C and medium was replaced every three days. Total mRNA was extracted using TRIzol reagent (Invitrogen, 15596026) according to the manufacturer's instructions. Total RNA was converted to cDNA using a ReverTra Ace™ qPCR RT Kit (TOYOBO, FSQ-201). RPL13A was used as an internal control. Briefly, the sequences of primers are listed below:

human BMP2 sense/antisense: 5′- TGACGAGGTCCTGAGCGAGT-3′/5′-AGGTGATAAACTCCTCCGTGGG-3′; human RUNX2 sense/antisense: 5′- ACTACCAGCCACCGAGACCA-3′/5′- ACTGCTTGCAGCCTTAAATGACTCT-3′; human OSTEOCALCIN (OCN) sense/antisense: 5′- CACTCCTCGCCCTATTGGC-3′/5′- CCCTCCTGCTTGGACACAAAG-3′; human cementum-derived attachment protein (CAP) sense/antisense: 5′- CTGCGCGCTGCACATGG -3′/5′- GCGATGTCGTAGAAGGTGAGCC-3′. Real- time PCR was performed with the Applied Biosystems QuantStudio® 3 Real-time PCR System (Thermo-Fisher Scientific), according to the manufacturer's instructions. For each PCR reaction, 5 µl of 1:10 diluted cDNA samples were used. For all PCR amplification reactions, the following parameters were used. After an initial incubation for 10 min at 95 °C, 45 cycles of denaturation at 95 °C for 10 s were followed by annealing for 10 s at 68 °C and elongation for 16 s at 72 °C. The final elongation was performed at 72 °C for 10 min. The samples were detected by SYBR Green (TOYOBO, QPK-201) incorporation into double stranded PCR products. The specificity of the reaction was tested by melting curve analysis. Standard curves built from 5 standard dilutions were used to determine the number of cDNA copies for each gene. The results were analysed using Quant Studio Design & Analysis Software (Thermo-Fisher Scientific). Gene expression was calculated using the delta-delta Ct method with RPL13A housekeeping gene, all from the same cDNA sample. The experiments were performed in triplicate. Groups were analysed with one-way ANOVA followed up with Tukey's multiple comparison tests in GraphPad Prism 9. Normal distribution of results was tested with Shapiro-Wilk test. Adjusted P value is reported in graphs according to New England Journal of Medicine guidelines: $P < 0.001$ (***), $P < 0.002$ (**), $P < 0.033$ (*), $P > 0.12$ (ns).

1.17 Preparation of fluorescently-labelled nanowires

For fluorescent dye labelling, 2.5 mg of pSiNs from stock were transferred in sequence to 2 ml of 0.5M HCl, DI Water and ethanol by centrifugation at 20,000 x g for 20 minutes, followed by supernatant removal and redispersion in the new solvent. To the ethanol suspension 2% of (3-Aminopropyl)triethoxysilane (APTES, Sigma-Aldrich) was added, and the suspension was incubated in a thermomixer at 800 rpm and 37 °C for 2 hours. The suspension was centrifuged at 20,000 x g and the supernatant was discarded. The nanowires were washed twice by redispersing into ethanol followed by centrifugation at 20,000 x g and removal of the supernatant. Nanowires were then dispersed in 4 ml of ethanol and 4 µl of 1 mM of AlexaFluor 568 NHS ester (ThermoFisher Scientific) in DMSO was added. The suspension was agitated in a thermomixer at room temperature for 4h. The suspension was centrifuged at 20,000 x g for 20 minutes and the supernatant was discarded. The nanowires were washed in ethanol three times as described above. Nanowires were stored in ethanol at 4 °C before using for up to two weeks.

1.18 Nanowire distribution following PDL injection

The mice used in this study were handled in accordance with UK Home Office Regulations project license P5F0A1579 and personal license IE6A6EC69. Experimental procedures were approved by the King's College Ethical Review Process. Wild-type mice (CD-1 background) were obtained from Jackson Laboratory. AlexaFluor 568 labeled pSiNs at 3.2 mg/ml in PBS were prepared by adding a known volume of stock Alexa-pSi suspension with known concentration in an Eppendorf tube, followed by centrifugation at 20 000 x g for 20 minutes, and drying for 30 min at 60 °C, and finally redispersion into appropriate PBS volume to obtain a 3.2 mg/ml concentration. Approximately 4 µl (12.8 µg of Alexa-pSi) of the solution was injected in three spots (distal, mesial and middle) around the periodontal ligament of the first molar of the wild-type mice and sealed with Tissue Adhesive (3M Vetbond, 1469SB). 24 hours post injection, mandibles were isolated and fixed in 4% paraformaldehyde (PFA) for 24-hours at 4 °C, washed in PBS and then embedded in OCT medium. 25 µm sections were cut using a cryostat (OTF5000, Bright Instruments) with a tungsten carbide blade. After washing the sections with PBS they were counterstained with DAPI (300 nM) in PBS. Immunofluorescence was visualized with a Leica TCS SP5 laser confocal microscope.

1.19 Evaluation of Wnt/β-catenin pathway activation following PDL injection

The mice used in this study were handled in accordance with UK Home Office Regulations project license P5F0A1579 and personal license IE6A6EC69. Experimental procedures were approved by the King's College Ethical Review Process. *Axin2*$^{LacZ/LacZ}$ mice were obtained from Jackson Laboratory. LipSiN-1.2% at 1.6 mg/ml in PBS were prepared by adding a known volume of stock LipSiN-1.2% suspension with known concentration in an Eppendorf tube, followed by centrifugation at 20,000 x g for 20 minutes, and drying for 30 min at 60 °C, and finally redispersion into appropriate PBS volume to obtain a 1.6 mg/ml concentration. Approximately 4 µl (6.4 µg of LipSiN-1.2%) of the solution was injected in three spots (distal, mesial and middle) around the periodontal ligament of the first molar of the wild-type mice and sealed with Tissue Adhesive (3M Vetbond, 1469SB). *Axin2*$^{LacZ/LacZ}$ mice mandibles were collected 24 hours after periodontal ligament injection. For X-gal staining, after a 5 h fixation in 0.2% glutaraldehyde in 0.1 mol/L phosphate buffer (pH 7.3), were washed three times with PBS. *Axin2*$^{LacZ/LacZ}$ mice mandibles were stained in PBS containing 5 mmol/L potassium ferrocyanide, 5 mmol/L ferricyanide, 2 mmol/L $MgCl_2$, 0.01% Na-deoxycholate, 0.02% NP-40, and 1 mg/mL X-gal. Staining was performed overnight at 37°C in the dark. The samples were washed with PBS and decalcified in 19% EDTA, pH 8 for 4 weeks and then were embedded in OCT, sectioned at 7 µm, and counterstained with Nuclear Fast Red.

1.20 Evaluation of LipSi activity in periodontal defect *in vivo*

1.20.1 Animals and surgical procedures

All animal experiments were performed under a protocol approved by the Institutional Animal Care and Use Committee of Peking University (LA2021-015).

50 Sprague Dawley rats (approx. weight 300 g acquired from Charles River Laboratories Inc., Beijing, China) were used and housed in cages in the following conditions: temperature (23 °C ± 1 °C), humidity (50% ± 10%), and a 12:12-hour light-dark cycle. The experimental animals were provided with water and standard laboratory chow. With 2 and 6 week time points, 5 different groups were designed: (1) control: surgery without any treatment, (2) LipSiN group: LipSi-1.2% nanowires (1.6 mg/ml) dispersed in Pluronic F-127 (15% in H_2O), (3) Lithium chloride (LiCl) group: LiCl (25mM, Sigma, SLBP6005V) dissolved in Pluronic F-127 (15% in H_2O), (4), bioglass group: bioglass (1.6 mg/ml) with 100% substitution of Na for Li, dispersed in Pluronic F-127 (15% in H_2O)[4], pSiN group: pSiNs (1.6 mg/ml) dispersed in Pluronic F-127 (15% in H_2O), and (5) Pluronic F-127 group: Pluronic F-127 (15% in H_2O). General anaesthesia was performed with sodium pentobarbital (40 mg/kg body weight, i.p.) for the initial periodontal defect creation surgery. Under the flushing, a periodontal fenestration defect (standardized with 3 mm in length, 3 mm in height and <1 mm in deep) was carefully prepared on the buccal side of the mandible with a low-speed turbine and a 3mm outer diameter treble (Hager & Meisinger GmbH, B42704). During the defect preparation, the saline was used for cooling. The muscle and skin incisions were sutured and closed respectively. Animals were euthanized using CO_2 for biopsy harvest.

1.20.2 μCT analysis

To evaluate the formation of new bones in different groups, the fixed mandibles were scanned using CT imaging (Siemens Medical Solutions, Knoxville) at 80 kV and 500 mA. The Inveon Research Workplace software (Siemens) was applied to calculate the ratio of bone volume/tissue volume (BV/TV) and bone mineral density (BMD) in the defect area according to a protocol provided by the manufacturer. Statistical significance was tested by Kruskal-Wallis non-parametric multivariate analysis followed by Dunn's multiple comparison post-hoc test. N = 5 independent biological samples.

1.20.3 Histological analyses and multiplex immunofluorescent staining

After CT scanning, rat mandibles were decalcified in 15% EDTA for 4 weeks, dehydrated in a graded series of ethanol and embedded in paraffin. Serial tissue sections with 7 µm in thickness were prepared from the transverse plane of the defect area, stained with hematoxylin-eosin (HE) and Trichrome Stain Kit (Connective Tissue Stain) (Abcam, ab150686), and observed under a light microscope (Carl Zeiss Inc., Germany). After Masson's trichrome staining, the blue color indicates the regenerated bone, collagen fibers, or osteoid, while the red color indicates the mature bone. For multiplex immunofluorescent staining. The 6 µm thick paraffin sections were deparaffinized in xylene and rehydrated in a series of graded alcohols. Antigen retrieval was performed in citrate buffer (pH = 6) by heating in a microwave (Sharp, R-331ZX) for 20 mins at 95 °C followed by a 20 mins cool-down period at room temperature. Multiplex fluorescence labelling was performed using TSA-dendron-fluorophores (NEON 9-color All round Discovery Kit for FFPE, Histova Biotechnology, NEFP950). In brief, endogenous peroxidase was quenched in 3% H_2O_2 for 20 mins, followed by application with blocking reagents for 30 mins at room temperature. Primary antibody was incubated for 2–4 hours in a humidified chamber at 37°C, followed by detection using the HRP-conjugated secondary antibody and TSA-dendron-fluorophores. Then, the primary and secondary antibodies were thoroughly eliminated by heating the slides in retrieval/elution buffer (Abcracker®, Histova Biotechnology, ABCFR5L) for 10 s at 95 °C using a microwave. In a serial fashion, each antigen was labelled with distinct fluorophores. The multiplex antibody panels applied in this study were as follows: Osterix (Abcam, ab209484); CD31 (Cell Signalling, 77699); α-Smooth Muscle Actin (α-SMA) (Cell signalling, 19245). After all the antibodies were detected sequentially, the slices were imaged using the confocal laser scanning microscopy platform Zeiss LSM880.

1.21 LA-ICP-MS measurements

Laser ablation inductively coupled plasma mass spectrometry (LA-ICP-MS) measurements were conducted using an Iridia laser (Teledyne Photon Machines, Bozeman, MT, USA) coupled to an Agilent 8900 triple quadrupole ICP-MS (Agilent, Santa Clara, CA, USA). Tuning of the instrument settings was performed using a NIST SRM 612 glass certified reference material (National Institute for Standards and Technology, Gaithersburg, MD, USA), optimizing for low laser-induced elemental fractionation through monitoring of $^{238}U^{+}/^{232}Th^{+}$ ratios, low oxide formation (<1%) monitoring $^{232}Th^{16}O^{+}/^{232}Th^{+}$ ratios, and high sensitivity for $^{59}Co^{+}$, $^{115}In^{+}$ and $^{238}U^{+}$. LA-ICP-MS images were acquired in a fixed dosage mode (14 shots), with a vertical and horizontal spatial resolution of 20 µm. A laser fluence of 0.7Jcm^{-1} was used with a rep-rate of 280Hz and a scan speed of 400 µm/s. Dwell times of ^{7}Li: 21s, ^{29}Si: 5.2s, ^{31}P: 1, ^{55}Mn: 8.8s, ^{57}Fe: 1s and ^{88}Sr: 1s were allocated. To correct for instrumental drift, a series of NIST 612 standard ablation scans were performed before and after each image. ICP-MS and positional data were reconstructed using the HDF-based Image Processing software (HDIP, Teledyne Photon Machines Inc., Bozeman, MT, USA). A bespoke pipeline written in Python (version 3), was used to generate elemental images from reconstructed data. Negative values, attributed to instrumental noise, were replaced with zeros. All LA-ICP-MS images are displayed using the same threshold to enable a direct visual comparison between samples.

2 Supplementary Figures

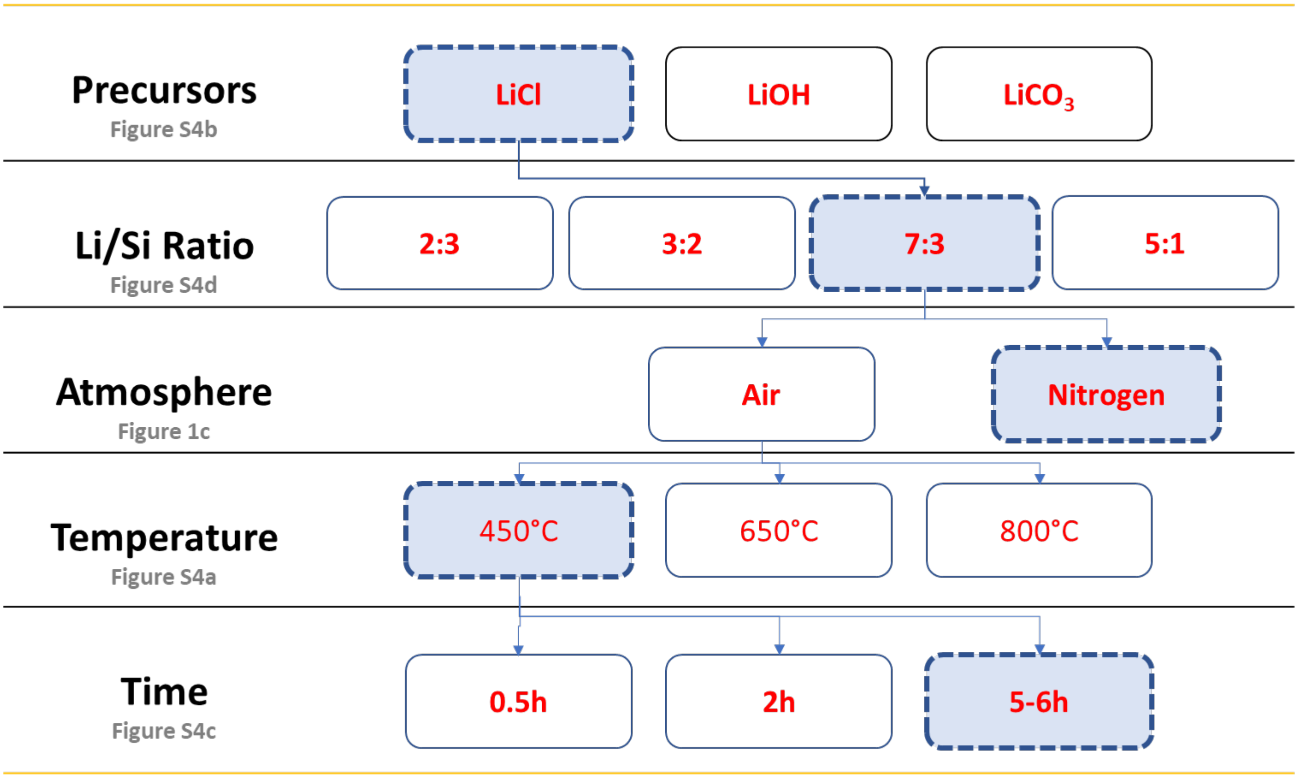

Supplementary Figure 1. Process parameter used for lithiation of porous Si (pSi) nanowires. Effect of different parameters on l ithiation process has been detailed in Figure 1c and S4.

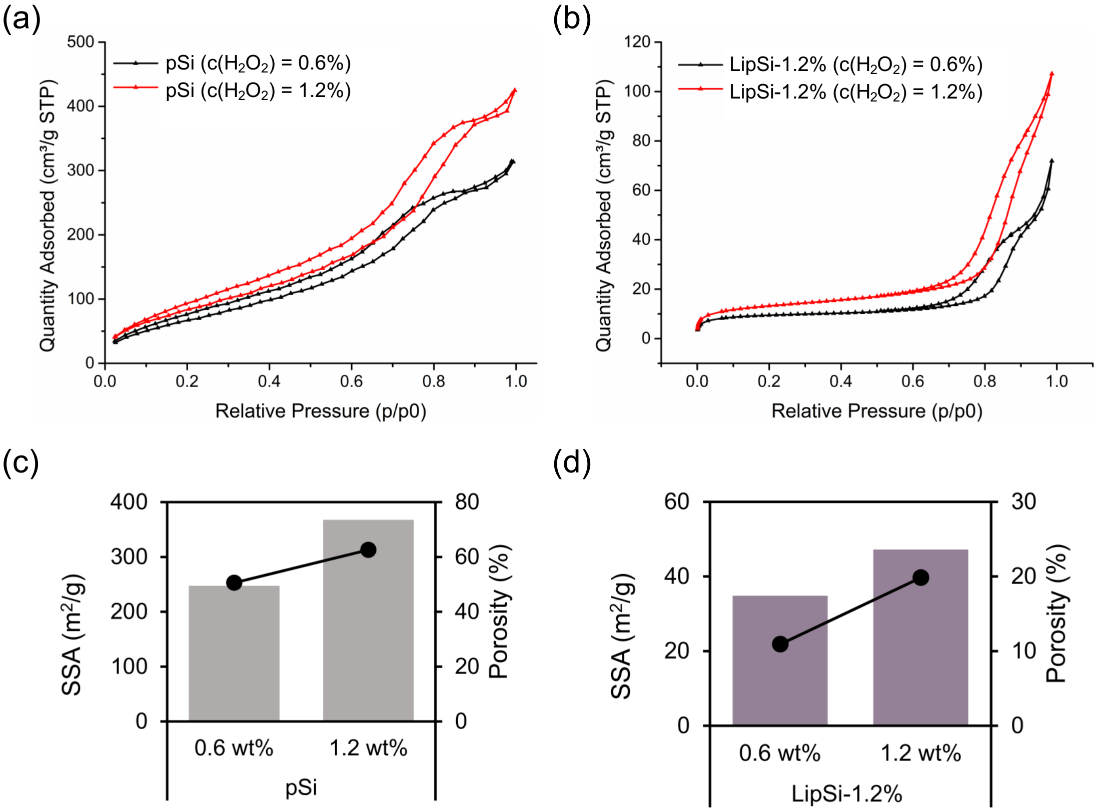

Supplementary Figure 2. Porosity of nanowires as a function of MACE etching condition and lithiation. Nitrogen sorption isotherms for pSi (a) and LipSi-1.2% (b) nanowires etched with two different hydrogen peroxide concentration during MACE shows evidence of mesoporosity for all materials considered. Calculation of specific surface area (SSA) and porosity for pSi (c) and LipSi-1.2% (d) nanowires as a function of H_2O_2 concentration during MACE.

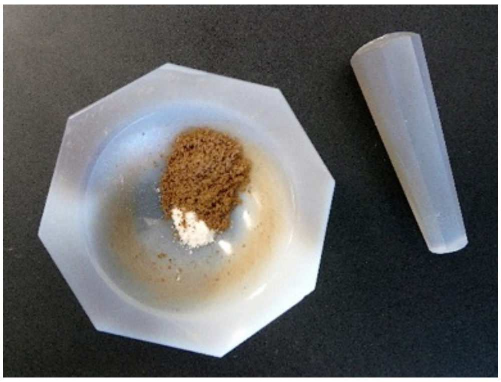

Supplementary Figure 3. Picture of the pSi nanowires before dry-mixing with LiOH in mortar.

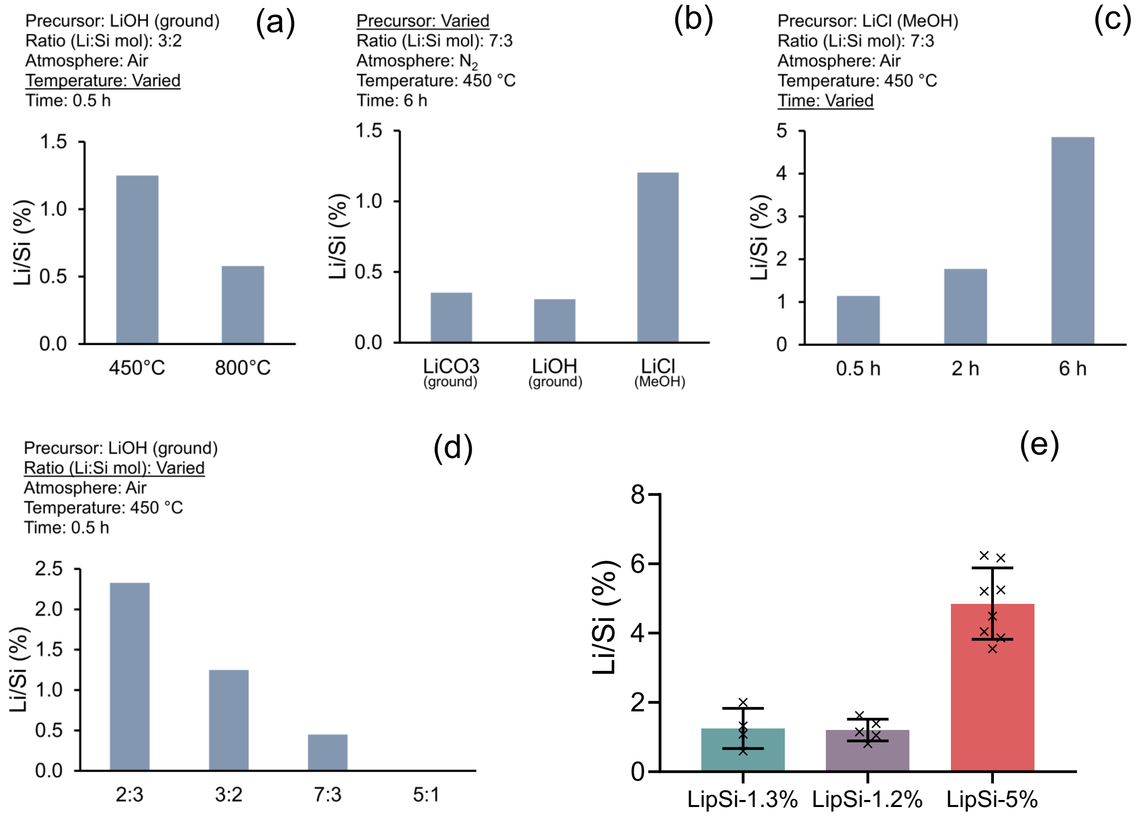

Supplementary Figure 4. Lithium content as a function of lithiation parameters. (a-d) Effect of lithiation parameters on lithium content of LipSiNs. Li/Si ratio measured by ICP-MS from LipSiNs. (a) Effect of temperature on lithiation with LiOH precursor. (b) Effect of precursor on lithiation at 450 °C under nitrogen flow. (c) Effect of time on lithiation with LiCl from methanoic solution. (d) Effect of Li:Si precursor ratio for LiOH precursor. (e) Repeatability of the lithiation process for LipSi 1.3% (4 batches), LipSi 1.2% (5 batches) and LipSi 5% (8 batches).

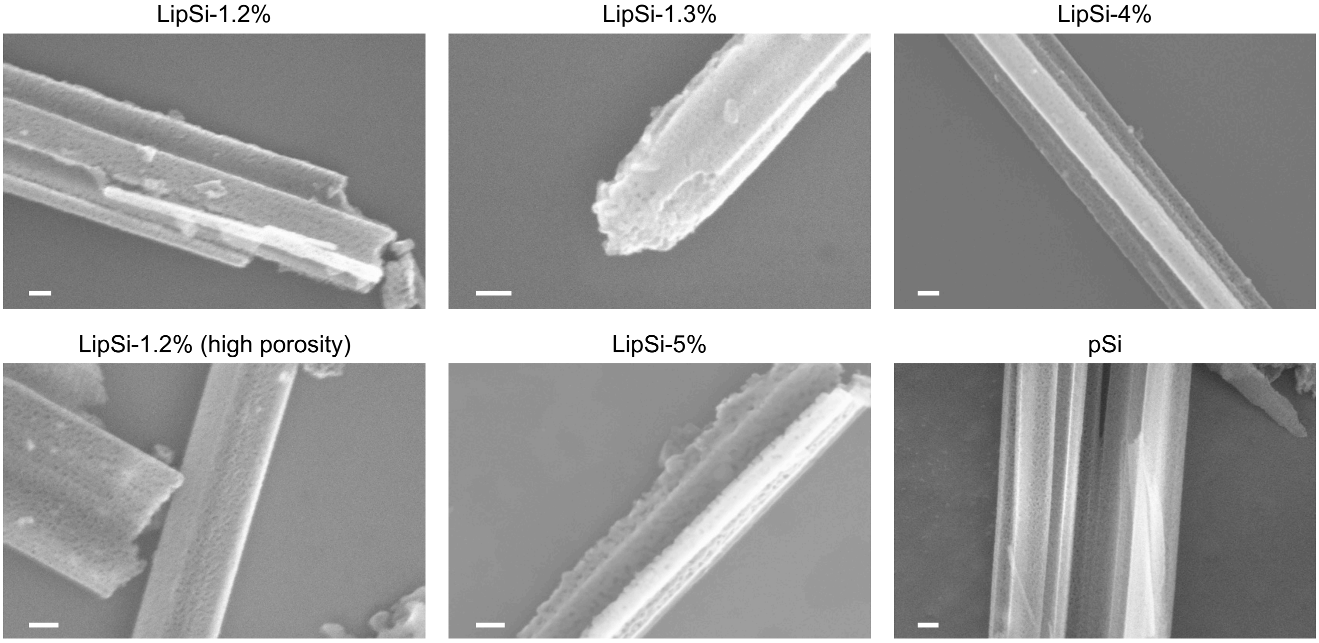

Supplementary Figure 5. Scanning electron micrographs highlighting the preserved mesoporous structure for pSi and LipSi nanowires. Scale bars 100 nm.

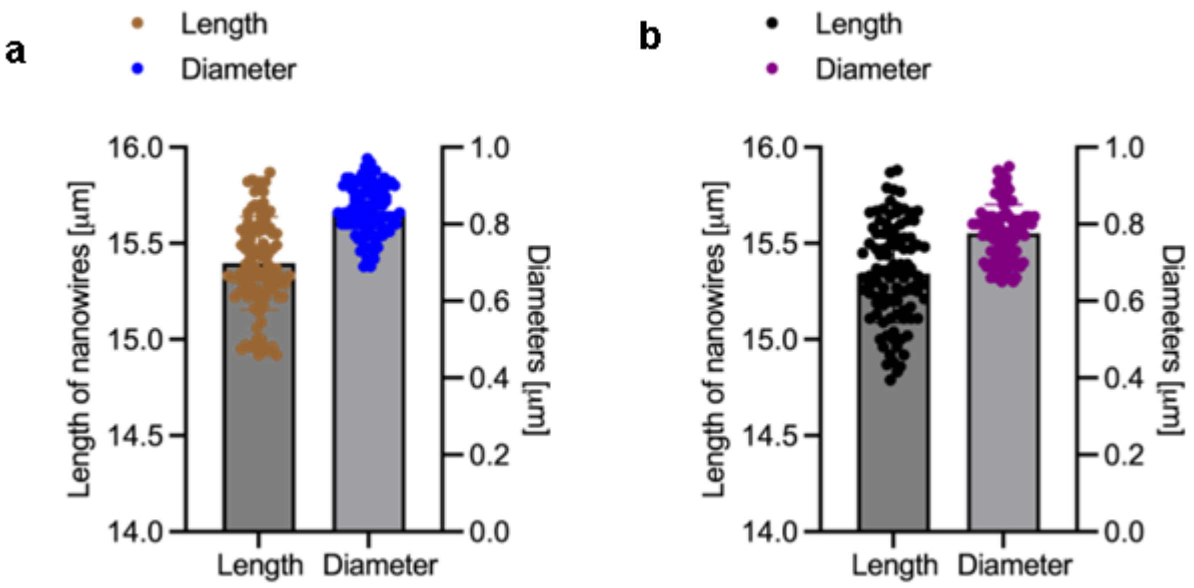

Supplementary Figure 6. Size distribution of (a) porous Si nanowires and (b) LipSi 1.2% nanowires. Data are presented as mean values ± S.D. 100 wires were measured per group from three independent samples.

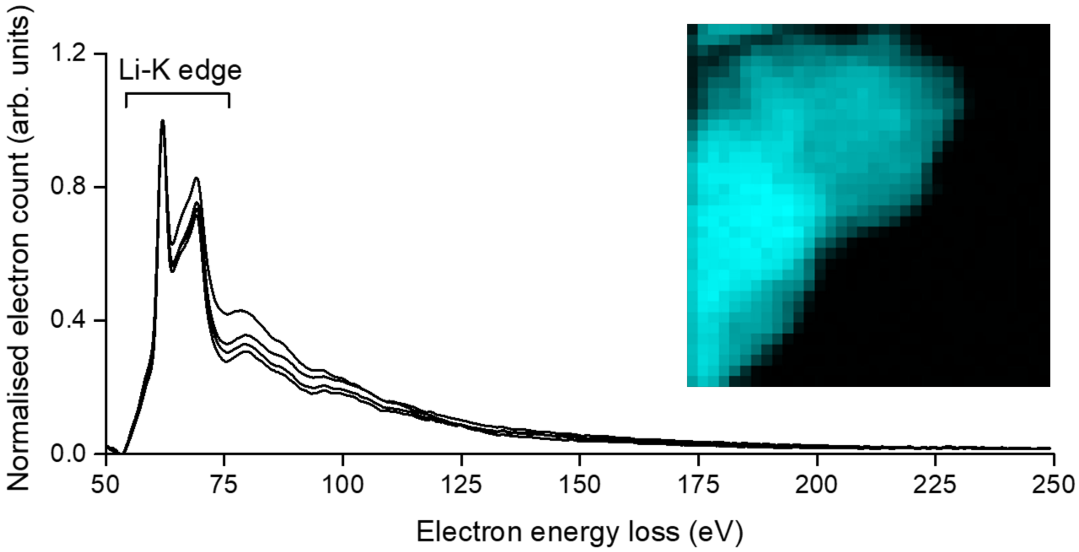

Supplementary Figure 7. EELS spectra and Li-K edge intensity map (60-65 eV) for the Lithium Fluoride standard.

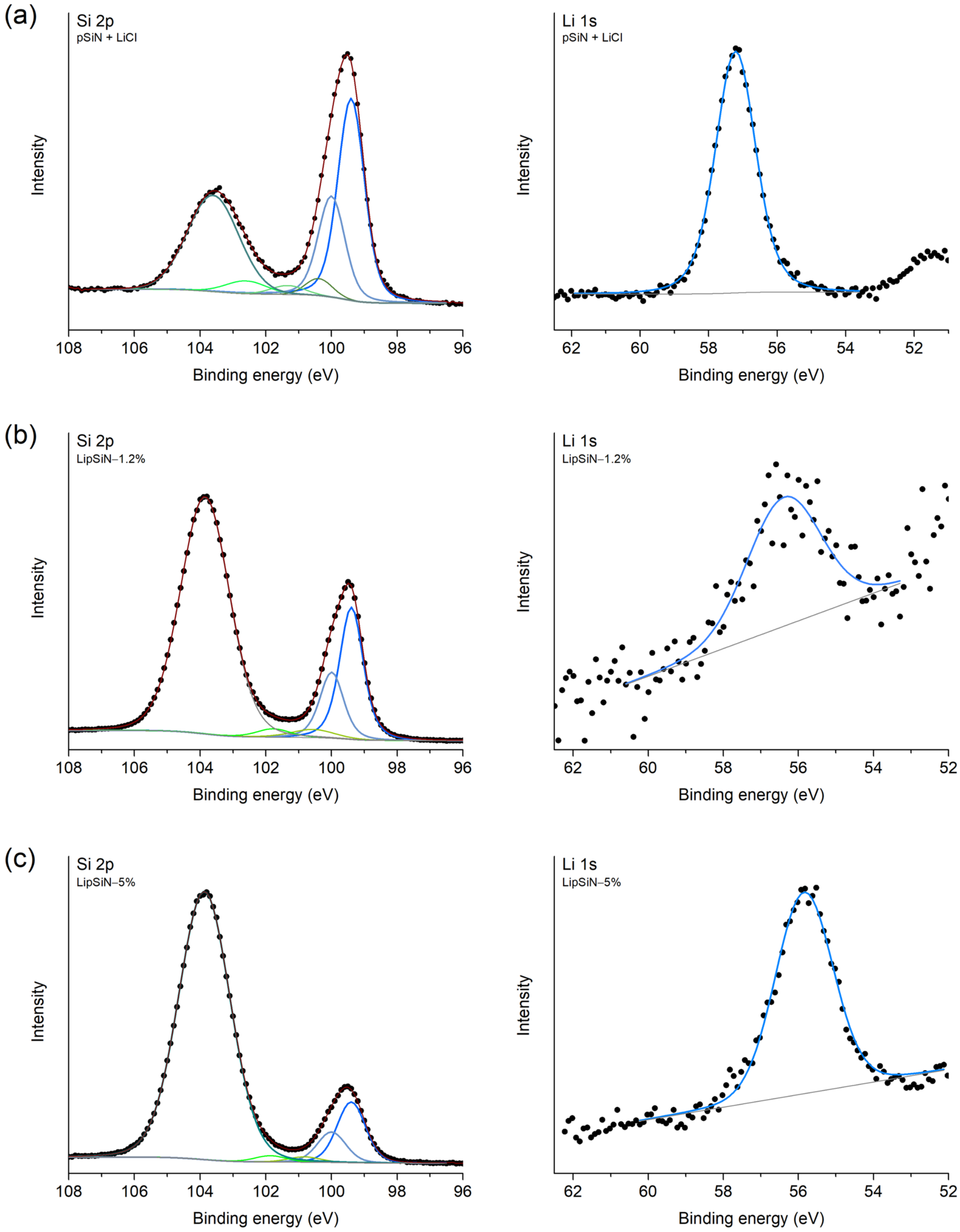

Supplementary Figure 8. Core level XPS spectra of Si *2p* and Li *1s* from (a) initial pSiN + LiCl mixture, and after thermal treatments at 450°C for (b) LipSiN-1.2% N_2 and (c) LipSiN-5% in air. With the initial pSiNs the suboxides (e.g. Si_2O, SiO, Si_2O_3) and the dioxide contribute approximately 8.5% and 32.5% of the Si *2p* signal, respectively. The N_2 annealed LipSiN-1.2% showed approximately 67% contribution from SiO_2 with additional 4% assigned to suboxides. LipSiN-5% due to its treatment in air has over 82% of the Si *2p* signal assigned to SiO_2 and slightly over 2% to suboxide compounds. The Li *1s* asymmetric Lorentzian peak was fitted using a linear background.

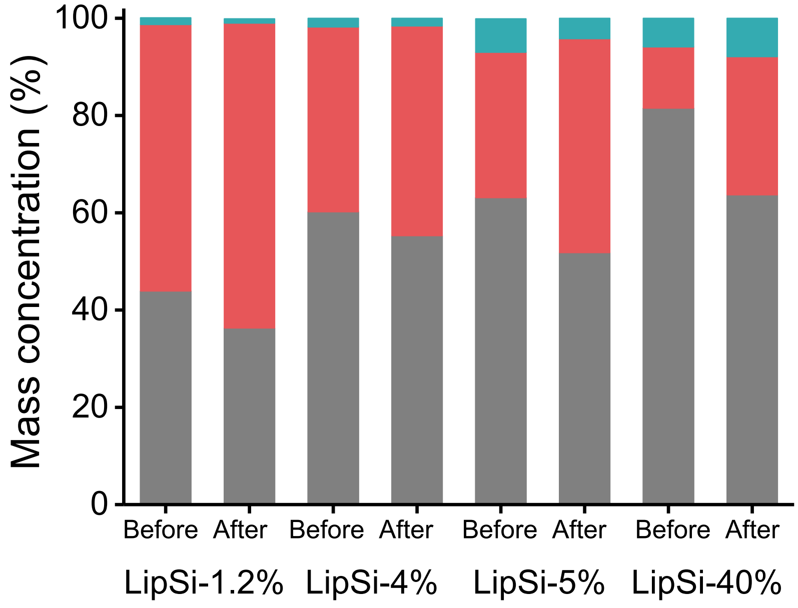

Supplementary Figure 9. Lithium (green), silicon (red) and oxygen (grey) relative concentrations quantified with XPS (transferred into mass concentrations).

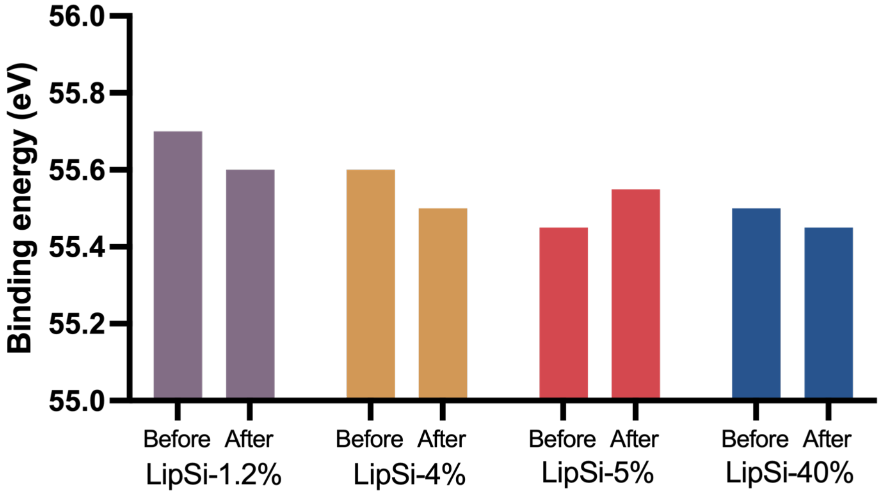

Supplementary Figure 10. Quantification of Li binding energy from X-ray photoelectron spectroscopy (XPS) analysis of the elemental composition of pSiNs and LipSiNs before and after surface sputtering by argon cluster beam. Analysis was performed on 1 sample.

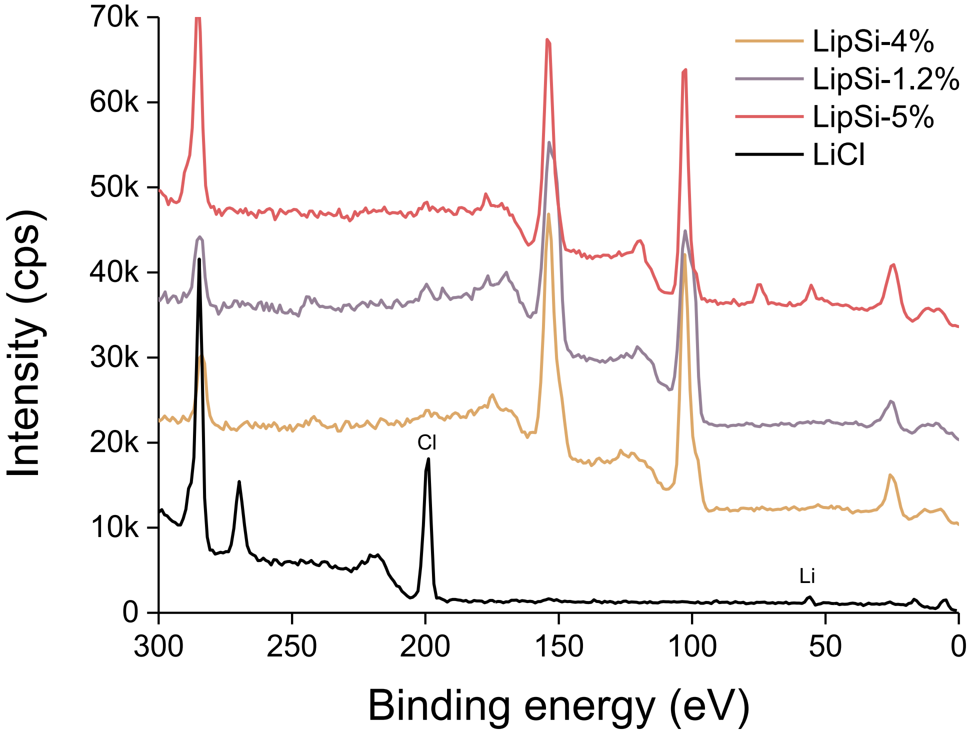

Supplementary Figure 11. XPS survey scan after argon etching does not show the presence of Cl.

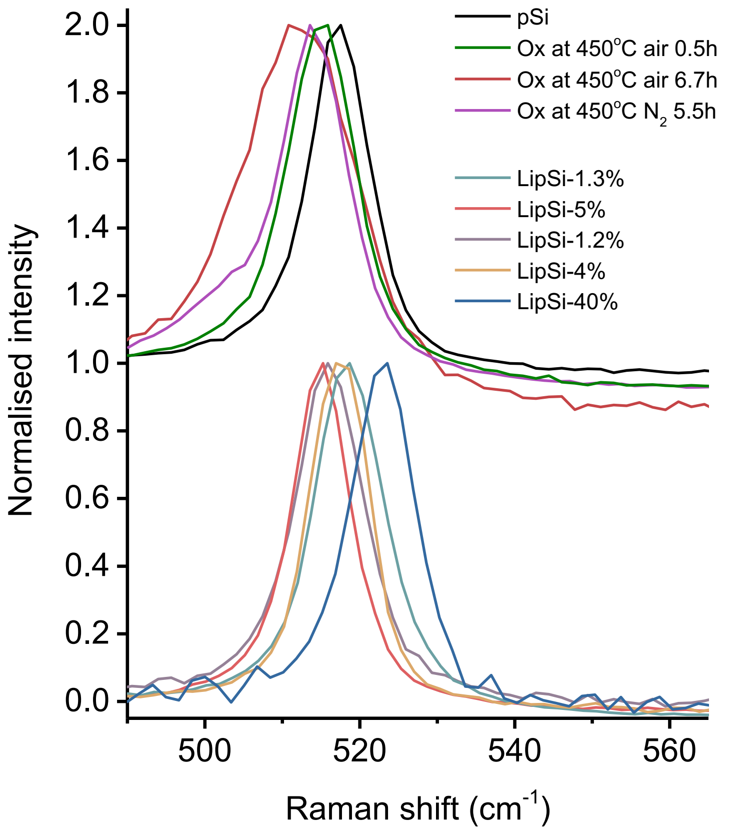

Supplementary Figure 12. Raman spectra for the Si peak of thermally treated pSi (above) showing red shift with respect to native pSi, and LipSiNs (below) showing blue shift compared to pSiNs thermally treated in comparable conditions.

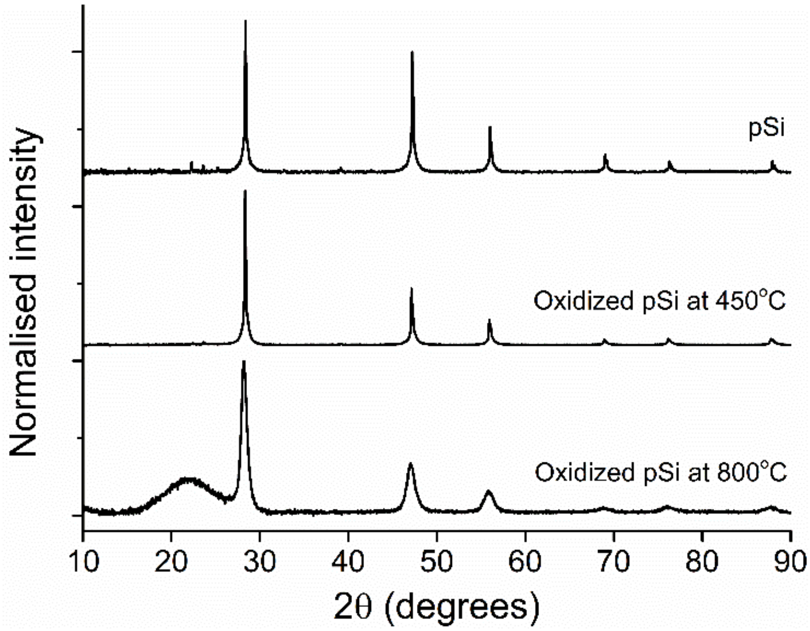

Supplementary Figure 13. XRD spectra for native pSi and oxidized pSi.

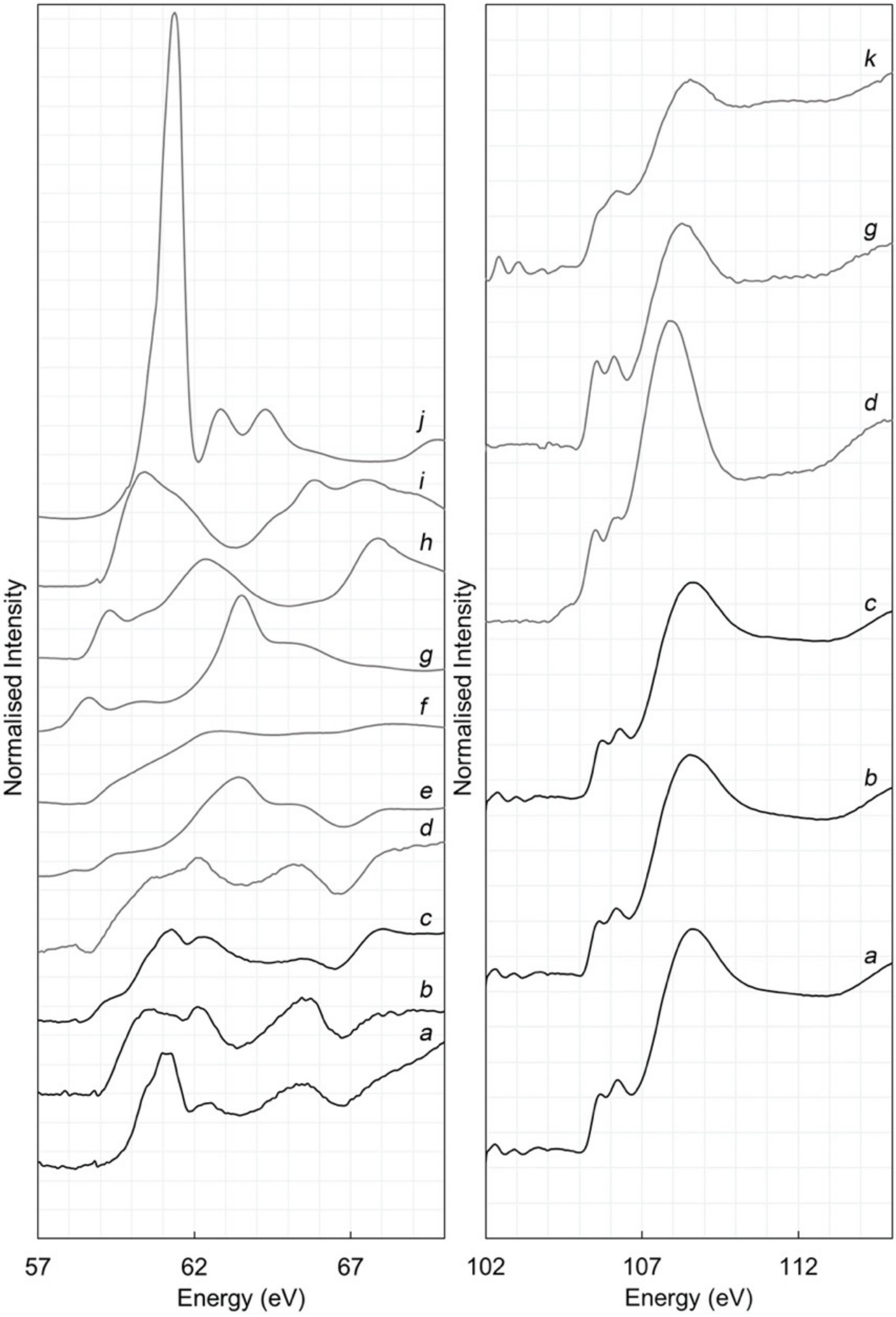

Supplementary Figure 14. XANES spectra on Lithium K-edge (left) and Silicon L-edge (right). Lithiated porous silicon samples labelled a, b, c represents LipSi-1.2%, LipSi-1.3% and LipSi-5% respectively. Standards are also shown included d; lithium bioactive glass, e; Li_2O, f; $Li_3C_6H_5O_7$, g; Li_xSi_y, h; Li_2CO_3, i; Li_2SiO_3, j; LiCl, k; Si wafer.

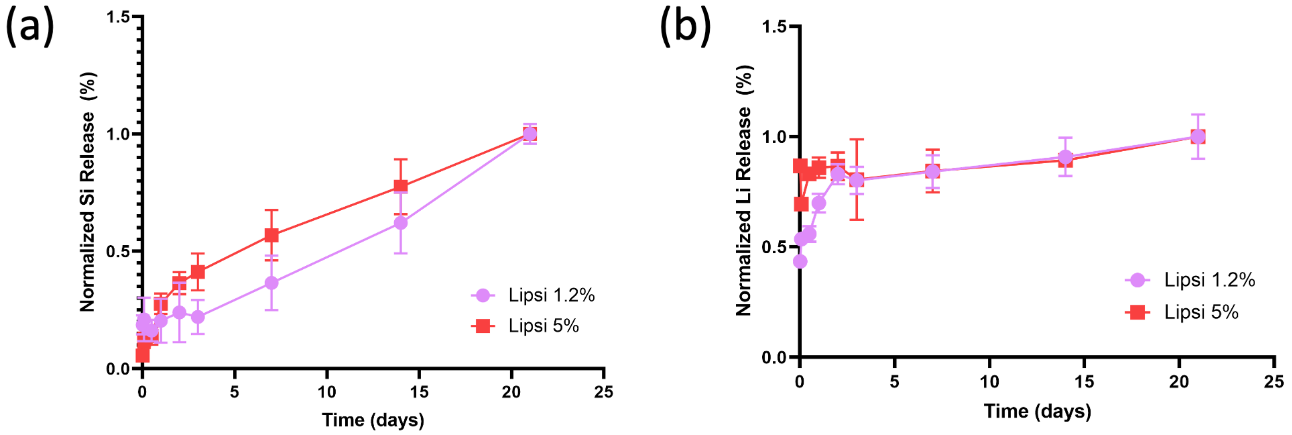

Supplementary Figure 15. (a) Si and (b) Li release profiles quantified by ICPMS from nanowires dissolving in simulated salivary fluid. Data are presented as mean values ± S.D. Data was collected from n = 3 independent measurements.

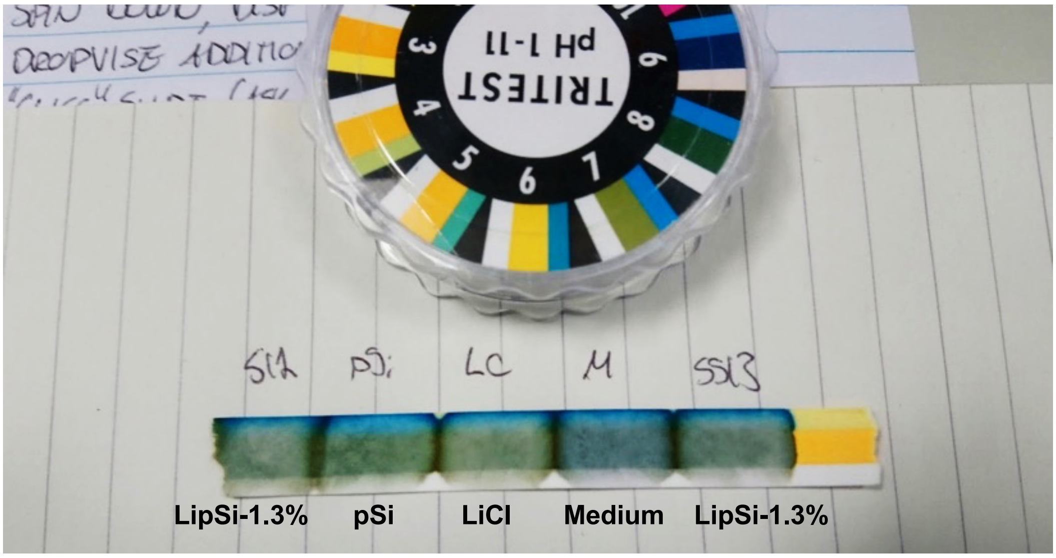

Supplementary Figure 16. pH test with LipSi-1.3% (left and right) and pSi conditioned media (800 µg/ml). LC: 25 mM LiCl and M: cell medium only.

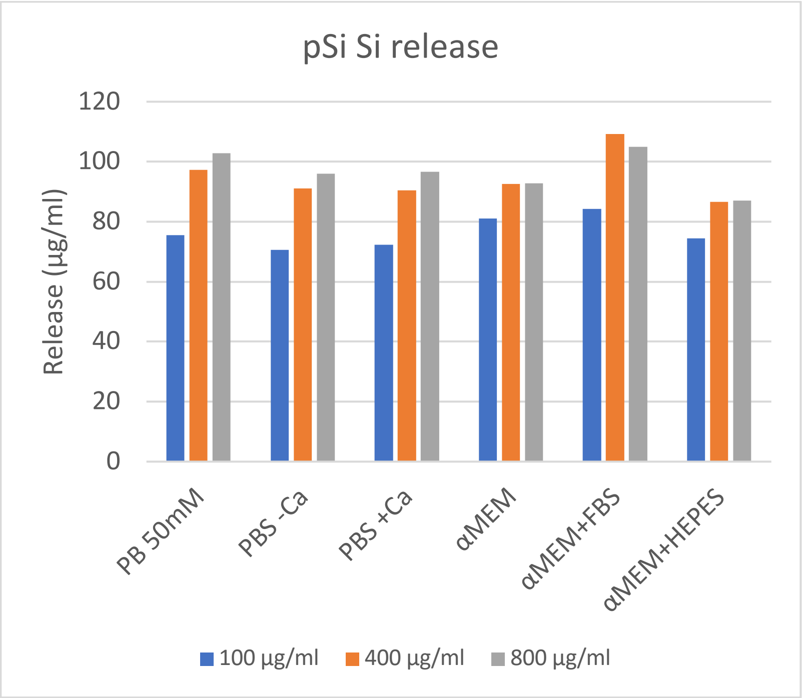

Supplementary Figure 17. Total Si released from pSi after conditioning with different concentrations and media for up to 4 days.

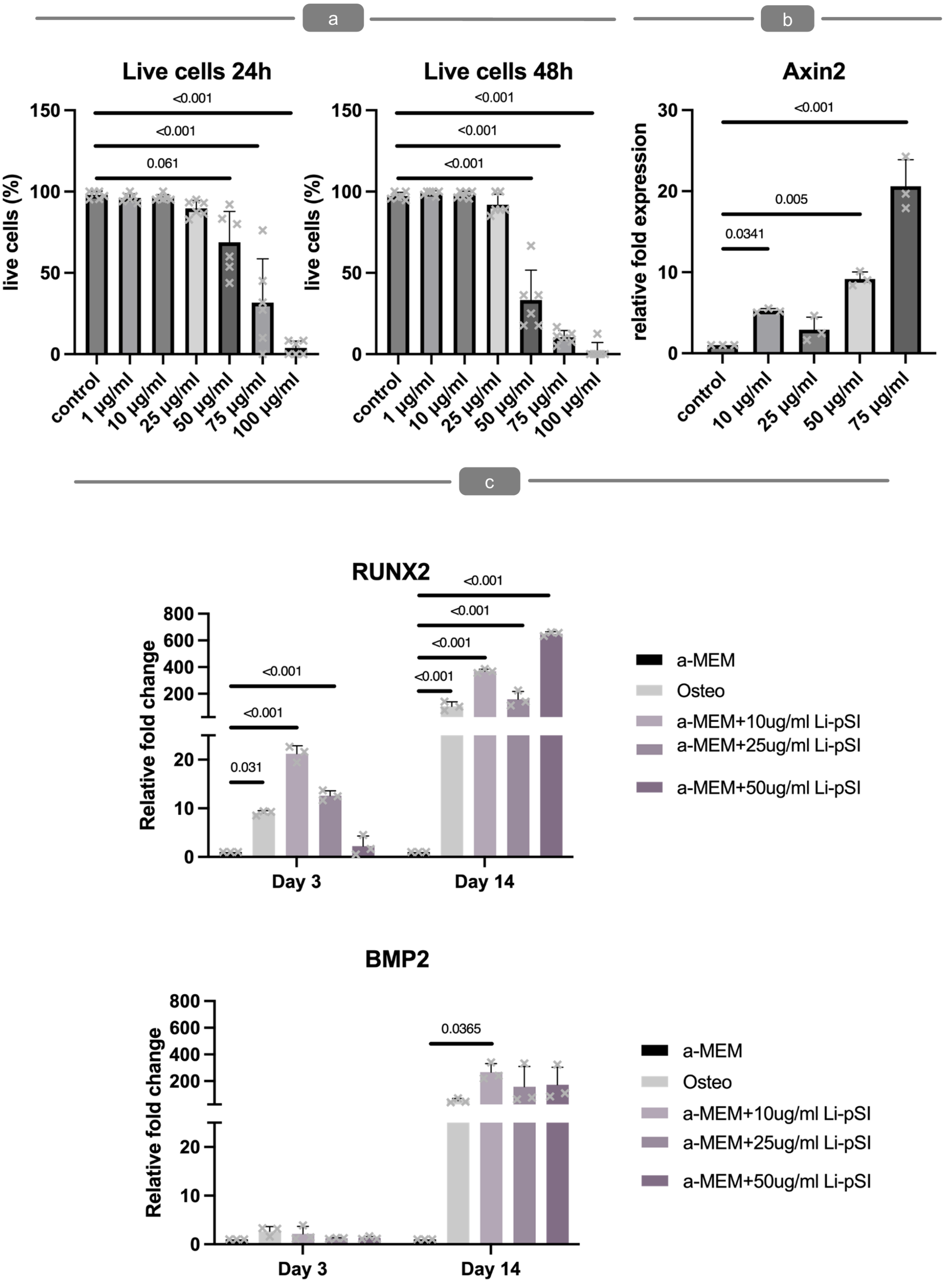

Supplementary Figure 18. (a) Cell toxicity test with 17IA4 cells after 48 hours and statistical analysis. (b) Relative fold change in the expression of *AXIN2* in 17IA4 cells after 24 hours culturing with different concentrations (75 µg/ml, 50 µg/ml, 25 µg/ml and 10 µg/ml) of LipSi-1.2%. (c) Relative fold change in the expression of osteogenesis genes (*RUNX2* and *BMP2*) after 3 and 14 days culturing with different concentrations (50 µg/ml, 25 µg/ml and 10 µg/ml) of LipSi nanowires. (a-c) Data are presented as mean values ± S.D. Statistical significance was tested with one-way ANOVA followed by followed by Dunnet's multiple comparison post-hoc test. Data was collected from n = 3 independent measurements.

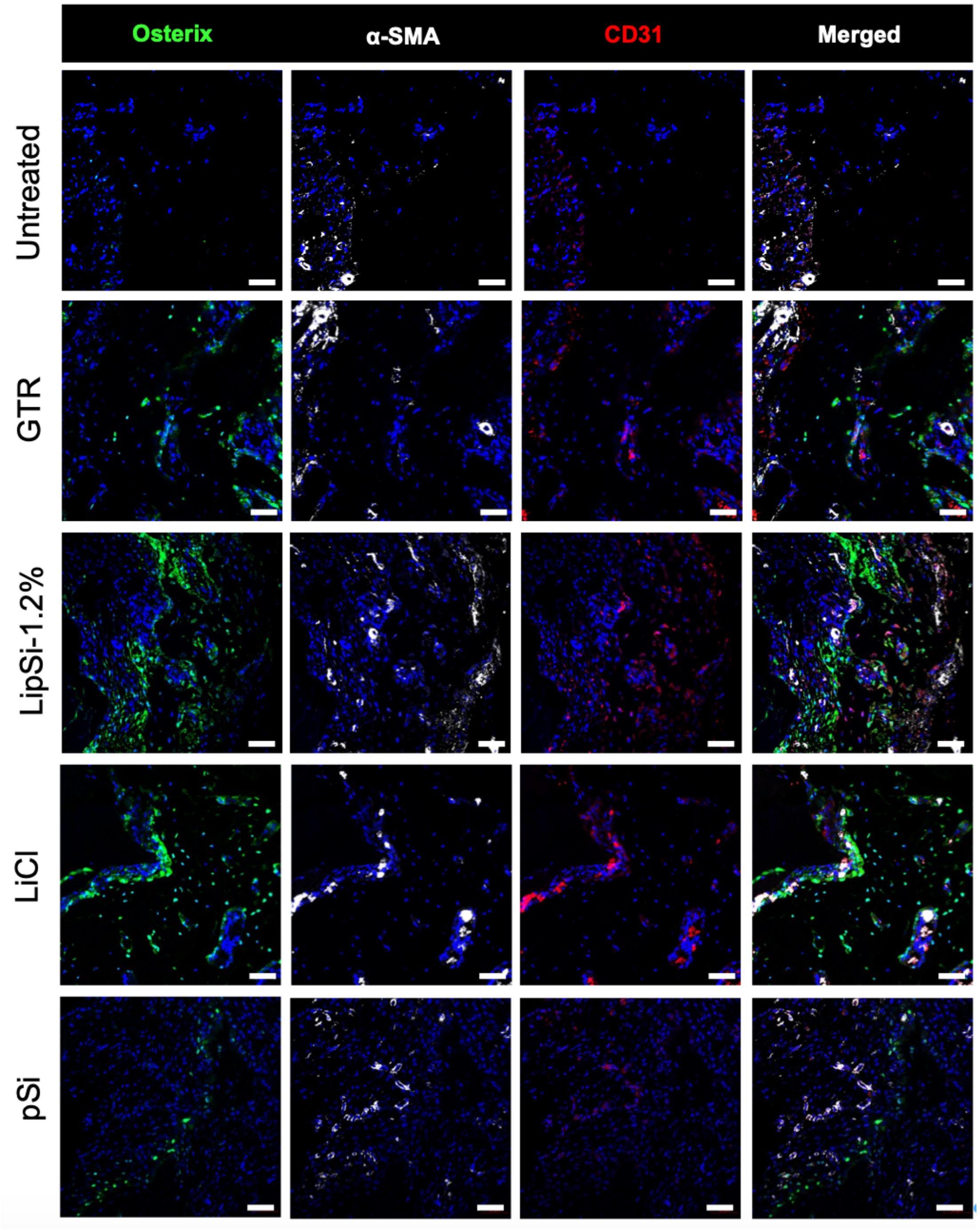

Supplementary Figure 19. Immunostaining at 2 week time point. Scale bars 50 µm. Analysis was performed on 4 animals.

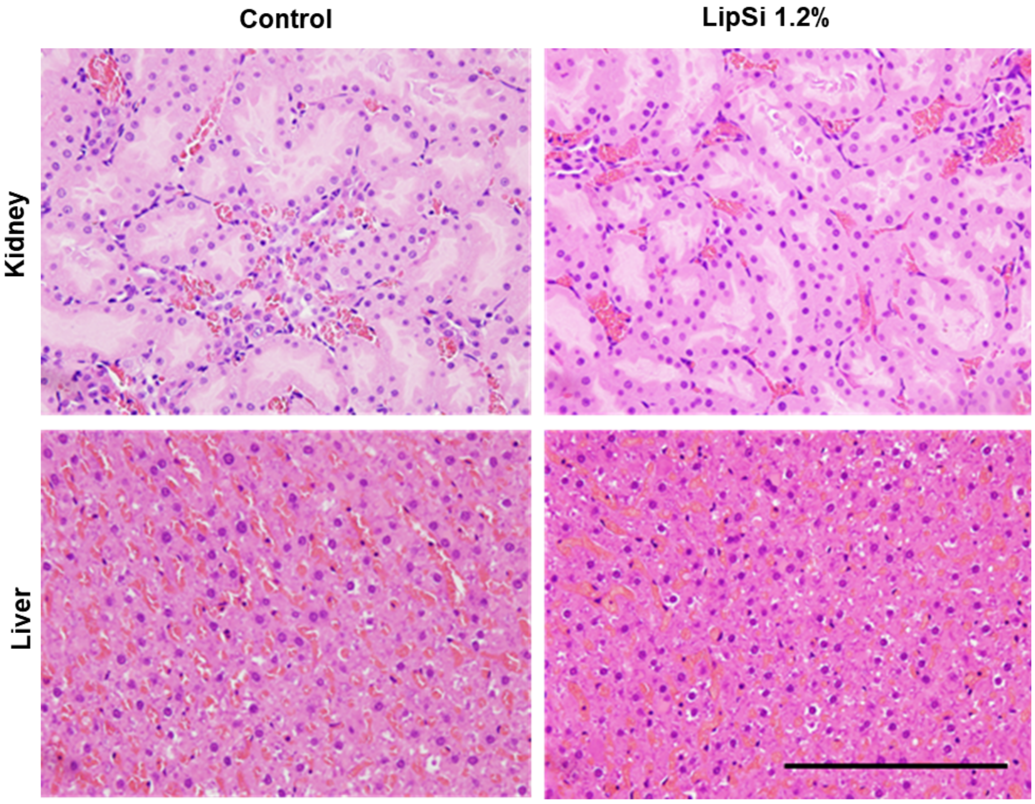

Supplementary Figure 20. HE staining of the kidney and liver of rats. Scale bars 200 µm.

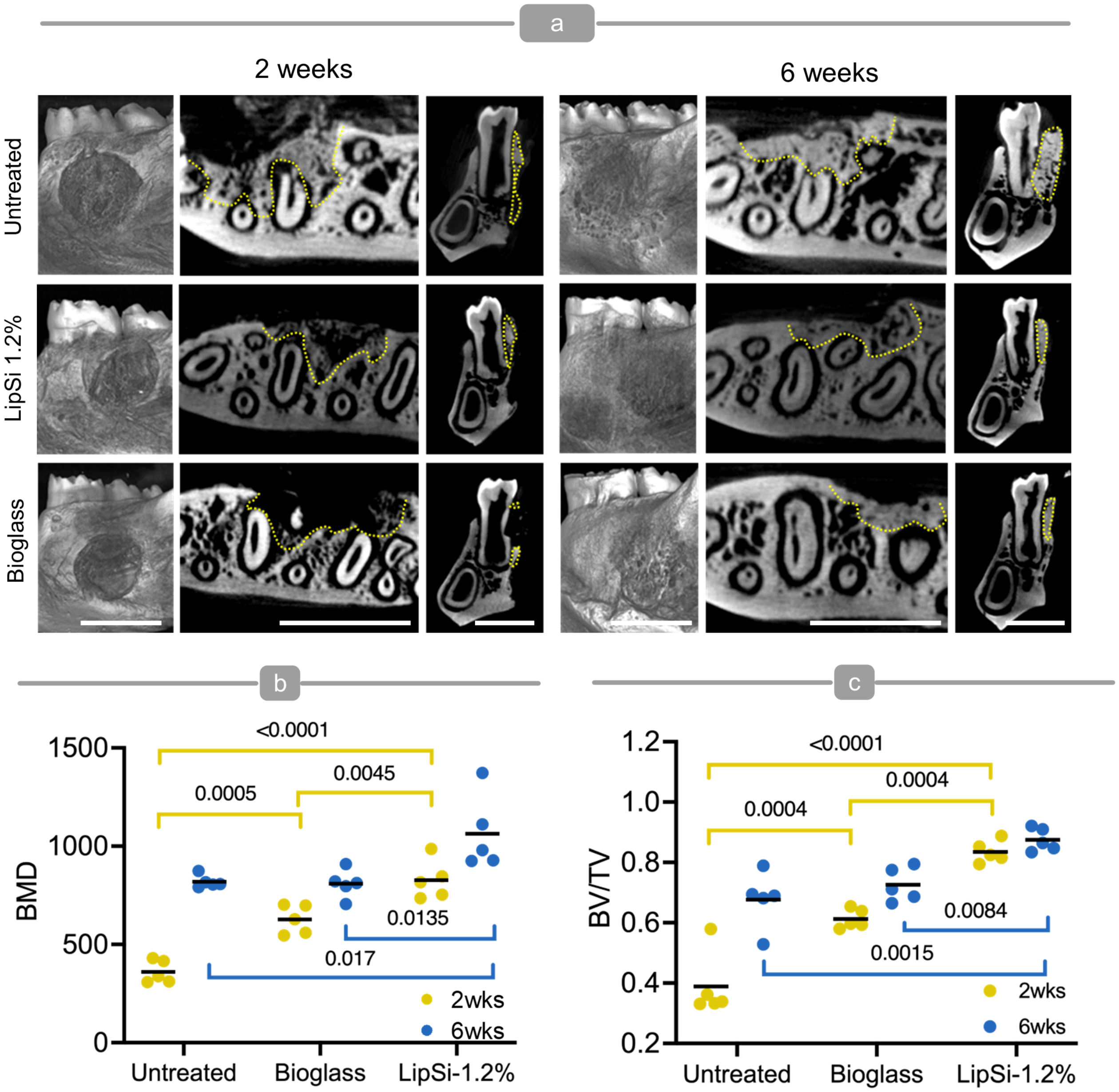

Supplementary Figure 21. Comparison of LipSi and lithium-substituted bioglass for alveolar bone regeneration. (a) µCT scans of rat mandibles showing regeneration of periodontal defects 2-weeks and 6-weeks post-operative with bioglass, and LipSi-1.2%; no treatment serves as baseline comparison. The dotted yellow line outlines the newly formed bone. Scale bar 3 mm. (b) µCT analysis for the quantification of bone volume over total volume (BV/TV) and (c) bone mineral density (BMD). (b,c) Data are presented as mean values ± S.D. N = 5 independent biological samples.

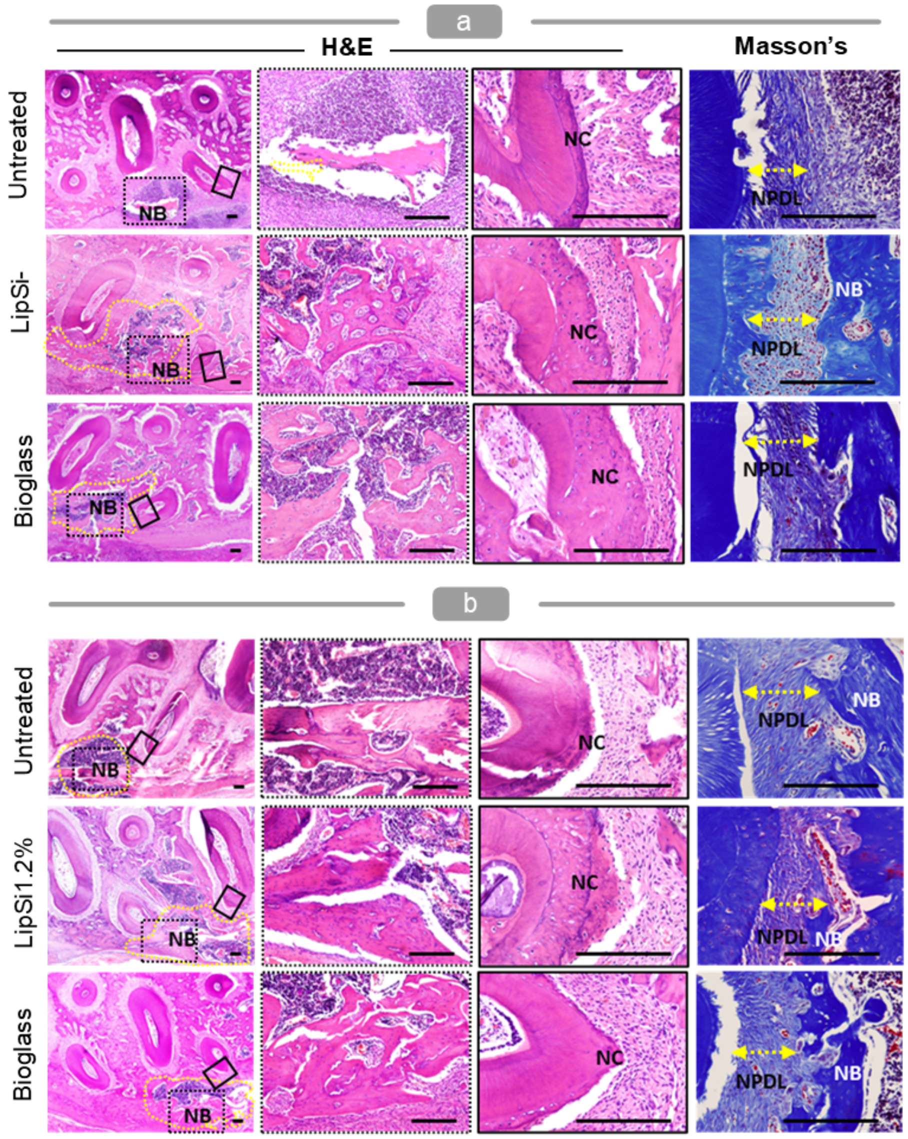

Supplementary Figure 22. Comparison of LipSi and lithium-substituted bioglass for periodontal regeneration. (a) Histological analysis of alveolar bone, cementum and periodontal ligament regeneration at 2 weeks and (b) 6-week post-operative. The three left panels show H&E staining, and the rightmost panel shows Masson's Trichrome staining. Leftside panel shows an overview of the periodontal defect and its regeneration. Within the leftside panel yellow dotted line indicates newly formed bone; black dotted line box locates the magnification panel used to visualise bone regeneration; black solid line box locates the magnification panel used to visualise cementum regeneration. Rightmost panel indicates periodontal ligament regeneration and its integration with bone and cementum. NB, new bone; NC, new cementum; NPDL, new periodontal ligament. Black arrow heads indicate the interface of the newly formed cementum on the root dentin. Scale bars 200 µm.

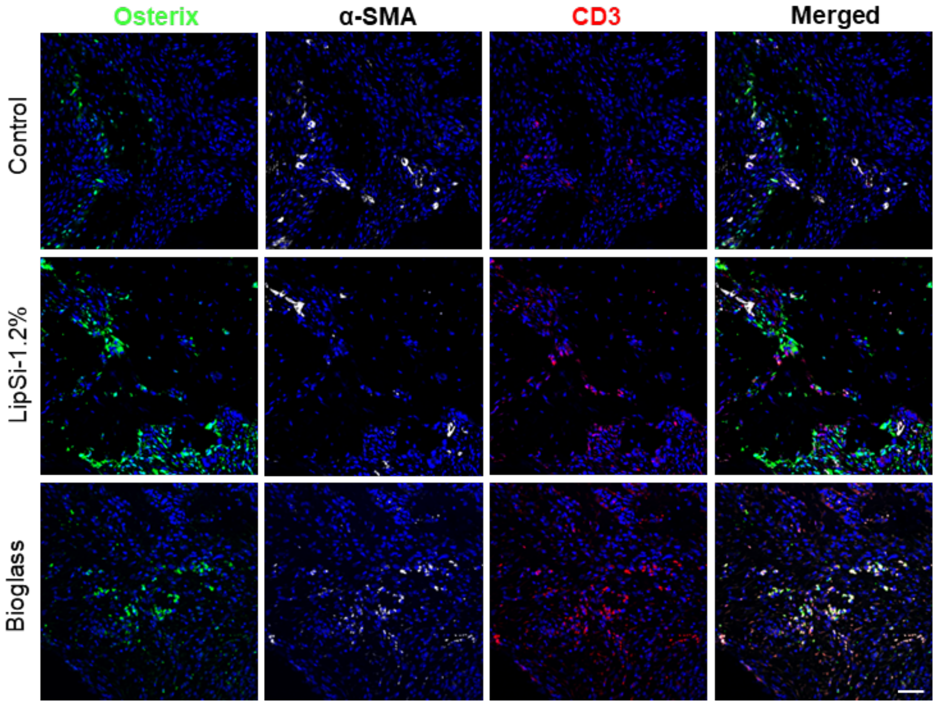

Supplementary Figure 23. Comparison of LipSi and lithium-substituted bioglass for periodontal regeneration. Co-immunofluorescence staining of histological slides with Osterix (green), α-SMA (white) and CD31 (red) in the area of the regenerated periodontium. Scale bars 50 µm.

References

1. Chiappini, C., Liu, X., Fakhoury, J. R. & Ferrari, M. Biodegradable porous silicon barcode nanowires with defined geometry. *Adv. Funct. Mater.* **20**, 2231–2239 (2010).

2. Jalkanen, T. *et al.* Selective optical response of hydrolytically stable stratified Si rugate mirrors to liquid infiltration. *ACS Appl. Mater. Interfaces* **6**, 2884–2892 (2014).

3. Seo, B. M. *et al.* Investigation of multipotent postnatal stem cells from human periodontal ligament. *Lancet* **364**, 149–155 (2004).

4. Tylkowski M, Brauer DS. Mixed alkali effects in Bioglass® 45S5. *J Non Cryst Solids*. 2013;376:175-181. doi:10.1016/j.jnoncrysol.2013.05.039
