# Supplementary material for: Lithiated porous silicon nanowires stimulate periodontal regeneration
Source: Nat Commun. 2024 Jan 12;15:487. doi: 10.1038/s41467-023-44581-5 (PMC10786831; doi:10.1038/s41467-023-44581-5)
Supplement: Supplementary file 3 — Reporting Summary [file 41467_2023_44581_MOESM3_ESM.pdf]

nature portfolio

Corresponding author(s): Ciro Chiappini DBPR; NCOMMS-22-50108-T

Last updated by author(s): Nov 6, 2023

Reporting Summary

Nature Portfolio wishes to improve the reproducibility of the work that we publish. This form provides structure for consistency and transparency in reporting. For further information on Nature Portfolio policies, see our Editorial Policies and the Editorial Policy Checklist.

Statistics

For all statistical analyses, confirm that the following items are present in the figure legend, table legend, main text, or Methods section.

n/a	Confirmed	
☐	☒	The exact sample size (*n*) for each experimental group/condition, given as a discrete number and unit of measurement
☐	☒	A statement on whether measurements were taken from distinct samples or whether the same sample was measured repeatedly
☐	☒	The statistical test(s) used AND whether they are one- or two-sided<br>*Only common tests should be described solely by name; describe more complex techniques in the Methods section.*
☐	☒	A description of all covariates tested
☐	☒	A description of any assumptions or corrections, such as tests of normality and adjustment for multiple comparisons
☐	☒	A full description of the statistical parameters including central tendency (e.g. means) or other basic estimates (e.g. regression coefficient) AND variation (e.g. standard deviation) or associated estimates of uncertainty (e.g. confidence intervals)
☐	☒	For null hypothesis testing, the test statistic (e.g. *F*, *t*, *r*) with confidence intervals, effect sizes, degrees of freedom and *P* value noted<br>*Give P values as exact values whenever suitable.*
☒	☐	For Bayesian analysis, information on the choice of priors and Markov chain Monte Carlo settings
☒	☐	For hierarchical and complex designs, identification of the appropriate level for tests and full reporting of outcomes
☒	☐	Estimates of effect sizes (e.g. Cohen's *d*, Pearson's *r*), indicating how they were calculated

Our web collection on statistics for biologists contains articles on many of the points above.

Software and code

Policy information about availability of computer code

Data collection	Data was collected using a wide range of commercial software associated to the instruments used. No custom code was developed for data collection.
Data analysis	GraphPad Prism 8.3.0, GraphPad Prism 9, Graphpad Prism 10, OriginPro 2022b, Excel 365

For manuscripts utilizing custom algorithms or software that are central to the research but not yet described in published literature, software must be made available to editors and reviewers. We strongly encourage code deposition in a community repository (e.g. GitHub). See the Nature Portfolio guidelines for submitting code & software for further information.

Data

Policy information about availability of data

All manuscripts must include a data availability statement. This statement should provide the following information, where applicable:

- Accession codes, unique identifiers, or web links for publicly available datasets
- A description of any restrictions on data availability
- For clinical datasets or third party data, please ensure that the statement adheres to our policy

All data presented in the manuscript is available from the authors upon request.

Human research participants

Policy information about studies involving human research participants and Sex and Gender in Research.

Reporting on sex and gender	This information is nor relevant to study and has not been collected.
Population characteristics	See above.
Recruitment	No human participants were recruited in this study.
Ethics oversight	See above.

Note that full information on the approval of the study protocol must also be provided in the manuscript.

Field-specific reporting

Please select the one below that is the best fit for your research. If you are not sure, read the appropriate sections before making your selection.

☒ Life sciences ☐ Behavioural & social sciences ☐ Ecological, evolutionary & environmental sciences

For a reference copy of the document with all sections, see nature.com/documents/nr-reporting-summary-flat.pdf

Life sciences study design

All studies must disclose on these points even when the disclosure is negative.

Sample size	Sample size was chosen according to nature of experiment based on the extensive experience of the investigators in order to ensure the accuracy and reproducibility of data. For synthesis of the material, physicochemical characterization and in vitro cell culture studies sample size was three replicates (n=3) and for animal experiment sample size was n=5 for each group.
Data exclusions	No data was excluded.
Replication	Experiments were done in replicates. Biological replicates were performed on different days with different cultures for all in vitro studies.
Randomization	Animals were randomly assigned to their respective group and tagged accordingly prior to establishing the defect model.
Blinding	The quantification of bone growth by microCT was performed blinded by a different researcher from the individual that performed the animal study and the scans.

Reporting for specific materials, systems and methods

We require information from authors about some types of materials, experimental systems and methods used in many studies. Here, indicate whether each material, system or method listed is relevant to your study. If you are not sure if a list item applies to your research, read the appropriate section before selecting a response.

Materials & experimental systems

n/a	Involved in the study	
☐	☒	Antibodies
☐	☒	Eukaryotic cell lines
☒	☐	Palaeontology and archaeology
☐	☒	Animals and other organisms
☒	☐	Clinical data
☒	☐	Dual use research of concern

Methods

n/a	Involved in the study	
☒	☐	ChIP-seq
☒	☐	Flow cytometry
☒	☐	MRI-based neuroimaging

Antibodies

Antibodies used	Osterix (Abcam, ab209484); CD31 (Cell Signalling, 77699); α-Smooth Muscle Actin (α-SMA) (Cell signalling, 19245).
Validation	The presence of CD31+ and Osterix+ osteoblasts within newly-formed bone used to validate the regeneration of vascularized bone using Lipsi formulation (described in this study). Presence of α-SMA-expressing cells, used to establish an α-SMA+ mediated regeneration.

Eukaryotic cell lines

Policy information about cell lines and Sex and Gender in Research

Cell line source(s)	Primary human periodontal Ligament stem cells (hPDL) for osteogenic differentiation were provided by the Oral Stem Cell Bank of Beijing, Tason Biotech Co. Ltd. The hPDL for cytotoxicity and Wnt activation were isolated at King's College London.
Authentication	The hPDLs from the Oral Stem Cell Bank of Beijing were characterised according to B-M Seo et al. The hPDLs from King's College were tested by three-lineage differentiation according to Angelova et al.
Mycoplasma contamination	The cells used in this study tested negative for Mycoplasma.
Commonly misidentified lines (See ICLAC register)	*Name any commonly misidentified cell lines used in the study and provide a rationale for their use.*

Animals and other research organisms

Policy information about studies involving animals; ARRIVE guidelines recommended for reporting animal research, and Sex and Gender in Research

Laboratory animals	Axin2 LacZ/LacZ mice and Sprague Dawley rats were used in this study.
Wild animals	The study did not involve wild animals.
Reporting on sex	This information has not been collected.
Field-collected samples	The study did not collected sample from the field.
Ethics oversight	The mice used in this study were handled in accordance with UK Home Office Regulations project license P5F0A1579 and personal license IE6A6EC69. Experimental procedures were approved by the King’s College Ethical Review Process. The experimental protocol used to study with Sprague Dawley rats was approved by the Institutional Animal Care and Use Committee of Peking University (LA2021-015), China.

Note that full information on the approval of the study protocol must also be provided in the manuscript.
